# Patterns and Mechanisms of Ancestral Histone Protein Inheritance in Budding Yeast

**DOI:** 10.1371/journal.pbio.1001075

**Published:** 2011-06-07

**Authors:** Marta Radman-Livaja, Kitty F. Verzijlbergen, Assaf Weiner, Tibor van Welsem, Nir Friedman, Oliver J. Rando, Fred van Leeuwen

**Affiliations:** 1 Department of Biochemistry and Molecular Pharmacology, University of Massachusetts Medical School, Worcester, Massachusetts, United States of America; 2 Division of Gene Regulation, Netherlands Cancer Institute, and Netherlands Proteomics Center, Amsterdam, The Netherlands; 3 School of Computer Science and Engineering, The Hebrew University, Jerusalem, Israel; 4 Alexander Silberman Institute of Life Sciences, The Hebrew University, Jerusalem, Israel; Adolf Butenandt Institute, Germany

## Abstract

Replicating chromatin involves disruption of histone-DNA contacts and subsequent reassembly of maternal histones on the new daughter genomes. In bulk, maternal histones are randomly segregated to the two daughters, but little is known about the fine details of this process: do maternal histones re-assemble at preferred locations or close to their original loci? Here, we use a recently developed method for swapping epitope tags to measure the disposition of ancestral histone H3 across the yeast genome over six generations. We find that ancestral H3 is preferentially retained at the 5′ ends of most genes, with strongest retention at long, poorly transcribed genes. We recapitulate these observations with a quantitative model in which the majority of maternal histones are reincorporated within 400 bp of their pre-replication locus during replication, with replication-independent replacement and transcription-related retrograde nucleosome movement shaping the resulting distributions of ancestral histones. We find a key role for Topoisomerase I in retrograde histone movement during transcription, and we find that loss of Chromatin Assembly Factor-1 affects replication-independent turnover. Together, these results show that specific loci are enriched for histone proteins first synthesized several generations beforehand, and that maternal histones re-associate close to their original locations on daughter genomes after replication. Our findings further suggest that accumulation of ancestral histones could play a role in shaping histone modification patterns.

## Abstract 2

Tracking of ancestral histone proteins over multiple generations of genome replication in yeast reveals that old histones move along genes from 3′ toward 5′ over time, and that maternal histones move up to around 400 bp during genomic replication.

## Author Summary

It is widely believed that chromatin, the nucleoprotein packaged state of eukaryotic genomes, can carry epigenetic information and thus transmit gene expression patterns to replicating cells. However, the inheritance of genomic packaging status is subject to mechanistic challenges that do not confront the inheritance of genomic DNA sequence. Most notably, histone proteins must at least transiently dissociate from the maternal genome during replication, and it is unknown whether or not maternal proteins re-associate with daughter genomes near the sequence they originally occupied on the maternal genome. Here, we use a novel method for tracking old proteins to determine where histone proteins accumulate after 1, 3, or 6 generations of growth in yeast. To our surprise, ancestral histones accumulate near the 5′ end of long, relatively inactive genes. Using a mathematical model, we show that our results can be explained by the combined effects of histone replacement, histone movement along genes from 3′ towards 5′ ends, and histone spreading during replication. Our results show that old histones do move but stay relatively close to their original location (within around 400 base-pairs), which places important constraints on how chromatin could potentially carry epigenetic information. Our findings also suggest that accumulation of the ancestral histones that are inherited can influence histone modification patterns.

## Introduction

In addition to the information encoded in DNA sequence, replicating cells can inherit epigenetic information, which refers to variable phenotypes that are heritable without an underlying change in DNA sequence. It is widely accepted that chromatin, the nucleoprotein packaging state of eukaryotic genomes, provides one potential carrier of epigenetic information. Although definitive proof that chromatin per se carries epigenetic information during replication exists in very few cases [1], genetic studies in numerous organisms have identified key roles for chromatin regulators in multiple epigenetic inheritance paradigms [2],[3].

The idea that chromatin structure carries epigenetic information poses a central mechanistic question—since chromosome replication involves dramatic perturbations to chromatin structure ranging from old histone displacement to widespread incorporation of newly synthesized histones, how can chromatin states be stably maintained? To understand the mechanism by which chromatin states could be inherited, it is necessary to understand the unique challenges posed by histone protein dynamics during replication [4]–[7]. First, histones must at least transiently dissociate from the genome during passage of the replication fork—if old histones carrying epigenetic information do not re-associate with daughter genomes at the location from which they came, this could lead to “epimutation,” analogous to DNA bases moving relative to one another during genomic replication. Second, it is unknown to what extent newly synthesized histones deposited at different loci differ in their covalent modification patterns. Finally, how old histones influence new histones, the basis for positive feedback, can be considered analogous to asking what the equivalent of base-pairing is during chromatin replication.

Classic radioactive pulse-chase studies demonstrated that, in bulk, maternal histones segregate equally to the two daughter cells [4],[8]–[10]. It is unknown, however, whether maternal histones remain close to the locus from which they were evicted by the replication fork or whether maternal histones are incorporated at preferred genomic loci in the two daughter genomes [5],[7],[11]. The extent of maternal histone dispersal affects the stability of epigenetic states in theoretical models of chromatin inheritance [12], making experimental determination of this parameter a key goal for epigenetics research.

To address these fundamental questions, we carried out a genetic pulse-chase with epitope-tagged histone H3 [13] to follow ancestral H3 for several cell divisions after removal of the ancestral tag. We find that old histone proteins do not accumulate at epigenetically regulated loci such as the subtelomeres but instead accumulate at the 5′ ends of long, poorly transcribed genes. As expected, old histones do not accumulate at loci exhibiting rapid histone turnover, but we also find that 3′ to 5′ movement of old histones along coding regions and histone movement during replication are required to explain the patterns of ancestral histone retention we observe. We estimate that maternal histones stay within ∼400 bp of their original location during replication, providing the first measure of this crucial parameter. Finally, we identify a number of factors that affect old histone localization, such as topoisomerase I and the H4 N-terminal tail, which both affect the 5′ bias in localization patterns. In contrast, CAF-1 mostly affects histone turnover at promoters. Together, these results provide a detailed overview of the movement of ancestral histones across multiple cell generations and identify a number of mechanisms that play a role in shaping the landscape of ancestral histone retention.

## Results

**Figure 1:**
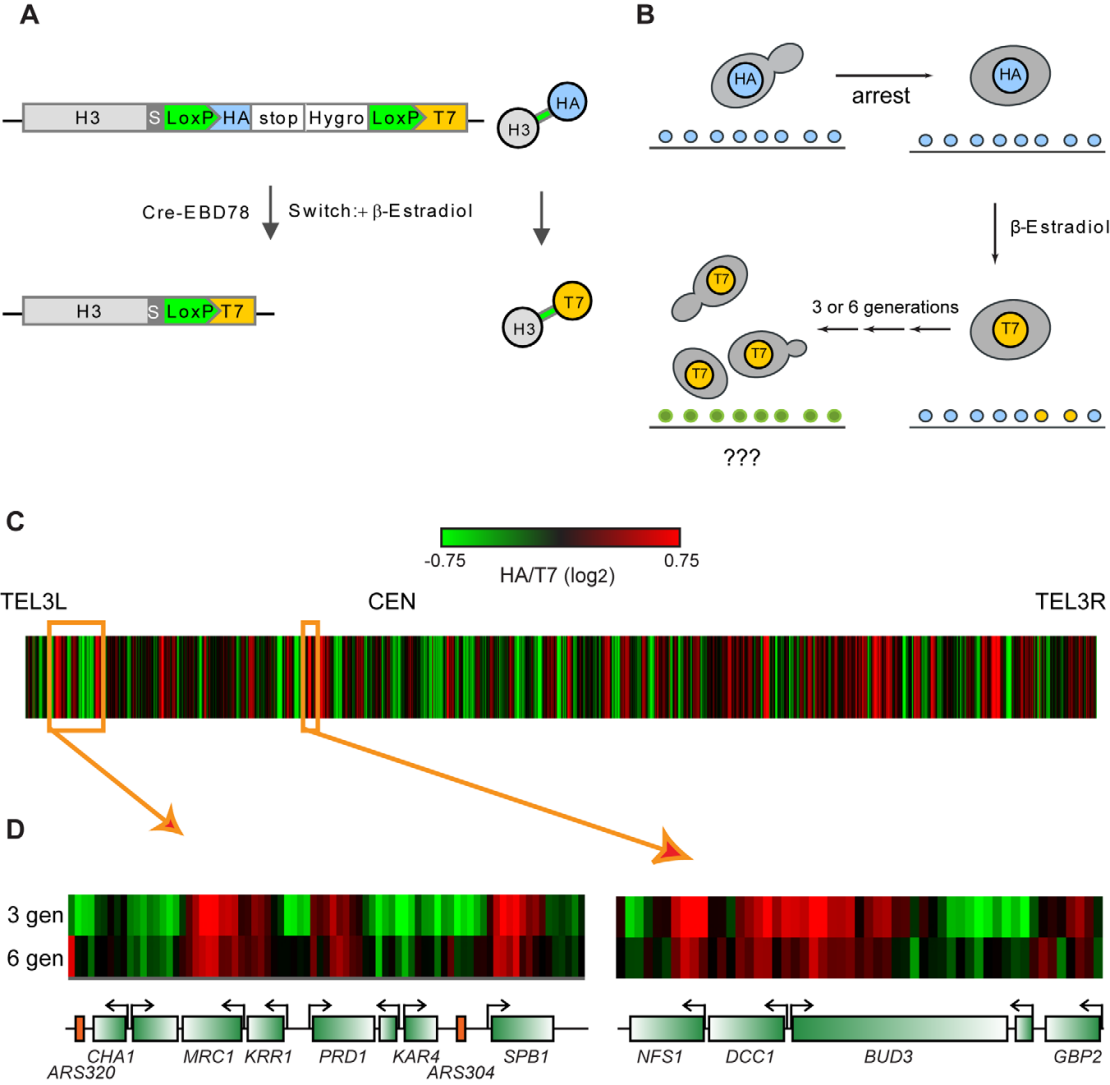
Overview of system for tracking ancestral histone proteins. (A) Recombination-based swapping of epitope tags on histone H3. Histone H3 is tagged at its endogenous locus with a C-terminal HA epitope tag surrounded by LoxP sites. Upon induction of Cre recombinase with β-estradiol, the HA tag is recombined out and H3 is left with a C-terminal T7 tag. (B) Experimental overview. Yeast carrying HA-tagged H3 are arrested by nutrient depletion, and the HA→T7 swap is induced by overnight incubation with β-estradiol. After the tag swap, yeast are released from arrest and HA and T7 tags are mapped across the genome at varying times post-release. (C) Chromosome III overview. HA/T7 ratios are shown as a heatmap across chromosome III at 3 generations after release. Notable in this view is a lack of accumulation of H3-HA at TEL3L or the silent mating loci. (D) Close-up views of two genomic loci. Data are shown as a heatmap for 3 and 6 generations after the tag swap.

To follow the movement of old histone proteins over multiple cell generations, we utilized a novel pulse-chase technique [13] to follow ancestral epitope-tagged histone H3 for several cell divisions after swapping epitope tags from H3-HA to H3-T7 (Figure 1A, B). We have previously described use of this technique to assay replication-independent H3 turnover in arrested cells and have shown that prior to recombination all cells carry the H3-HA, and that recombination is 98% efficient in cells that are not dividing due to nutrient deprivation (Figure S1). Unlike inducible p*GAL*-based systems for measuring replication-independent histone dynamics [14]–[17], here the epitope-tagged histone is under the control of its endogenous promoter, avoiding potential artifacts of H3/H4 misexpression [18] on histone dynamics throughout the cell cycle.

We used MNase-ChIP [15],[19] for the HA and T7 tags after recombination but before release into the cell cycle, and 3 and 6 generations after releasing yeast into the cell cycle [13]. This material was hybridized to tiling microarrays covering 4% of the yeast genome [20], and HA/T7 ratios of normalized HA and T7 signals were computed for the 3 and 6 generation data (Figure 1C, D). Since HA is eliminated via recombination leaving new H3-T7, high HA/T7 ratios indicate loci enriched for ancestral histone H3. Surprisingly, many of the highest HA/T7 levels were associated with coding regions (discussed below).

Overall, HA/T7 patterns are consistent at 3 and 6 generations, but the dynamic range of HA/T7 enrichment diminished from 3 to 6 generations (Figure 1D, Figure S2). This is an expected consequence of the fact that ∼1%–2% of cells do not recombine the HA tag away (Figure S1)—since the amount of ancestral H3 is decreasing by at least 2-fold in each generation, the relative contribution of the ∼2% of cells still expressing H3-HA will increase over time, with this genomic background eventually competing with the real signal from increasingly rare ancestral H3 (<2% of total H3 after 6 doublings).

### Ancestral Histones Are Retained Over Long, Poorly Transcribed Genes

To extend our analyses to the entire genome, we carried out deep sequencing of HA and T7 libraries. HA- and T7-tagged H3 were immunoprecipitated after the tag swap but before release from arrest (0 generations), after release into a G2/M cell cycle block, and at 1, 3, and 6 generations after release. Sequencing reads were mapped to the yeast genome, normalized for read count, and HA/T7 ratios were computed genome-wide. These data correlated well with our microarray data, and we further validated these measurements by q-PCR at *SPA2* and *BUD3*, two genes which both exhibit high and low HA/T7 ratios at their 5′ and 3′ ends, respectively (Figure S3).

In previous work, we and others [13],[15]–[17],[21]–[23] showed that there is a partial correlation between transcription levels and replication-independent histone dynamics. To understand how transcription might affect multigenerational histone retention in our system, we aligned all yeast genes by their transcription start site (TSS) and clustered genes (K-means, K = 5) based on the pattern of the 3-generation HA/T7 ratios along the gene body (Figures S4, S5, Table S1). We observed a striking enrichment of H3-HA just downstream of the 5′ ends of genes (typically peaking around the +3 nucleosome). One exception to the 5′ pattern described is found in one cluster of short genes with uniformly low H3-HA levels (Figure S4, Cluster 1), which is enriched for GO categories (such as protein translation) related to high gene expression levels. In contrast, long genes were generally associated with higher levels of ancestral H3 (see for example Cluster 5).

**Figure 2:**
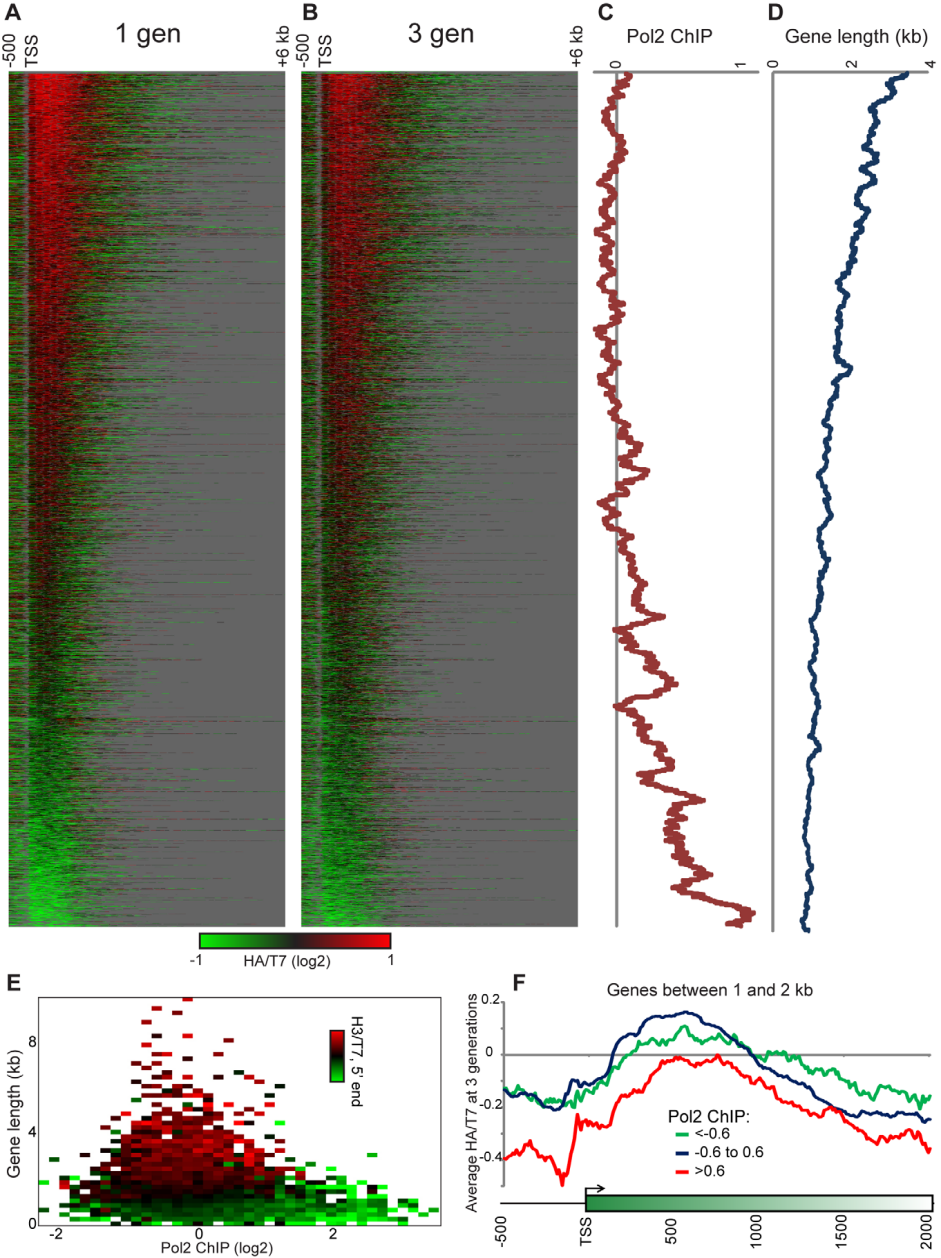
Ancestral H3 molecules accumulate at the 5′ ends of long, poorly transcribed genes. (A–B) Heatmap of sites of ancestral H3 accumulation. Genes are aligned by TSS (indicated), and Log_2_ HA/T7 ratios are indicated as a heatmap. Genes are ordered by the median HA/T7 ratio over the 5′-most 1 kb at 3 generations. Grey over coding regions indicates missing data; grey downstream of genes indicates sequence downstream of the 3′ end of the gene to show gene length. Accumulation of ancestral histones at the 5′ ends of genes peaks around the +3 nucleosome, as expected given that the +1 and +2 nucleosomes are generally subject to high rates of replication-independent H3/H4 replacement [15],[17]. (C) An 80 gene sliding window average of Pol2 ChIP levels [75] for genes ordered as in (A–B), showing that genes with low levels of ancestral H3 retention are highly transcribed. (D) 80 gene sliding window average of gene lengths, showing that genes with high levels of ancestral H3 retention tend to be long. (E) The median HA/T7 ratio over the 5′ end of genes (1 kb) was calculated for all genes, and median values of this retention metric are shown for groups of genes ordered by transcription rate (*x*-axis) and gene length (*y*-axis). While these are not independent—highly expressed genes tend to be short—for a given gene length genes transcribed at higher levels exhibit low HA retention levels. This is true mostly of genes shorter than 3 kb, which encompasses the majority of yeast genes. (F) Average HA/T7 ratios (Log2) for genes between 1 and 2 kb, broken into high (red), low (green), and intermediate (blue) transcription rates.

To better visualize these trends, we sorted genes by the extent of ancestral H3 retention after 3 generations (Figure 2A–B). Retention of ancestral histones correlates both with low expression levels and with longer genes (Figure 2C–D, Figure S6). While it is the case that longer genes tend to be expressed at lower levels than short genes (Figure 2E), these factors are partially independent here—even when we focus on genes of 1–2 kb length, we still observe the correlations between ancestral histone retention and low expression (Figure 2E–F, and see below). Interestingly, in both microarray and sequencing datasets we found that epigenetically repressed loci such as the silent mating loci and subtelomeres [24],[25] did not preferentially accumulate ancestral histone proteins (Figure 1C, Figure S7)—analysis of both unique and repetitive subtelomeric genes showed similar H3-HA retention patterns to euchromatic genes of similar length and expression. This was not a consequence of silencing defects in our strains, as they showed efficient mating (unpublished data).

**Figure 3:**
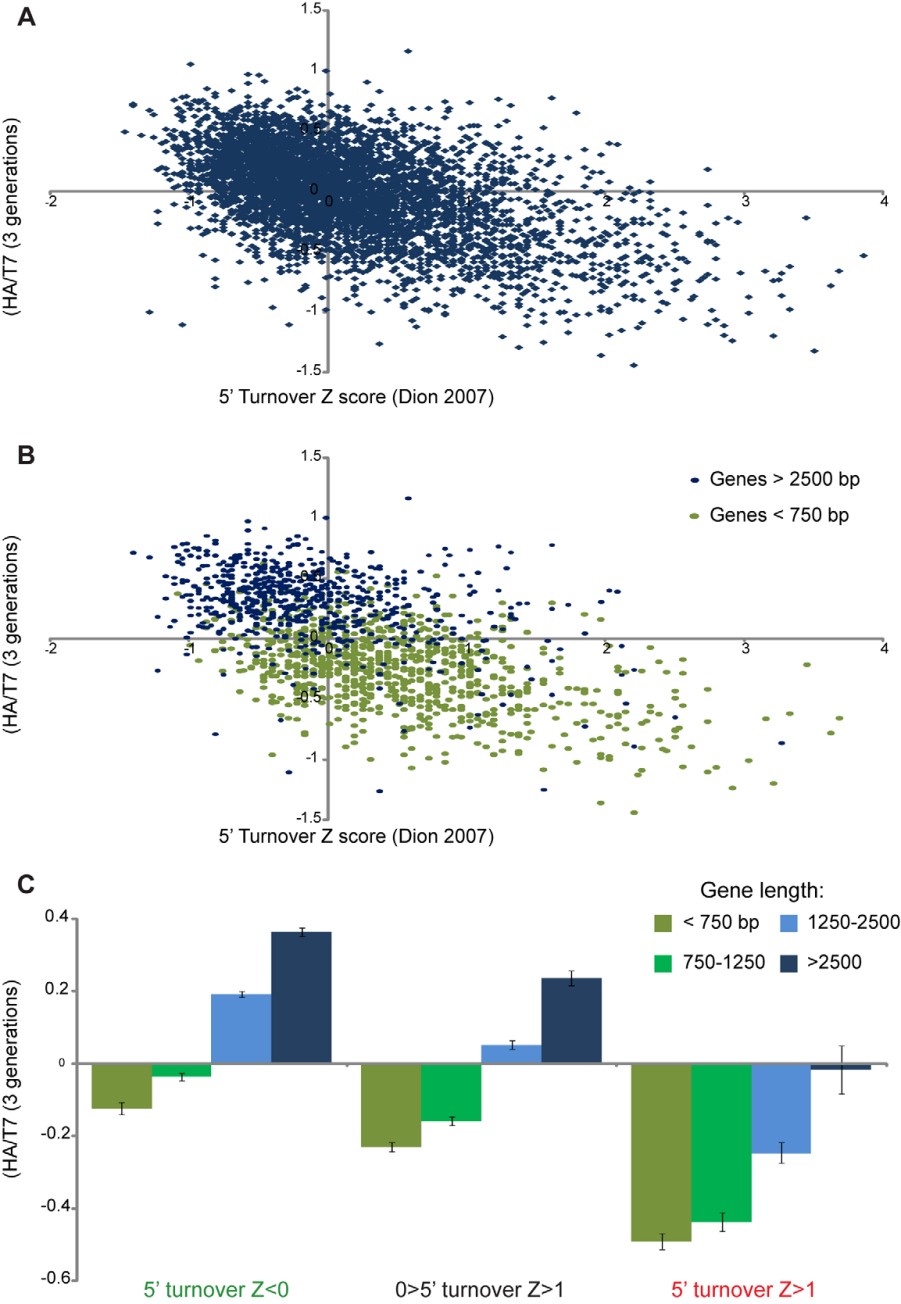
H3 retention anticorrelates with replication-independent turnover in a gene length-dependent manner. (A) Scatterplot of ancestral H3 retention (median Log2 HA/T7 for the 5′ 1 kb, *y*-axis) versus replication-independent turnover (Dion et al. [15], Z score, *x*-axis). (B) HA retention is plotted against 5′ H3 turnover as above but with short and long genes plotted separately. For a given level of H3 turnover, ancestral retention is greater at longer genes. (C) Averages of the 5′ HA/T7 retention parameter (median HA/T7 for the 5′-most 1 kb) are shown for genes broken into different length and 5′ turnover groups. For all turnover levels, longer genes retain more H3-HA than do shorter genes.

What properties of short or highly transcribed genes might lead to loss of ancestral histones? Replication-independent histone replacement occurs most rapidly over intergenic regions and over the coding regions of highly transcribed genes [15],[17],[21],[23], the converse of the pattern of ancestral H3 retention we observe. Indeed, ancestral histone retention is broadly correlated with “cold” regions of low H3/H4 turnover (Figure 3A). Importantly, however, for a given level of H3/H4 turnover, ancestral H3 retention varied significantly—retention at a given nucleosome was better correlated with the average turnover rate of several surrounding nucleosomes than with the immediate turnover rate (see, for example, Figure 3B–C). This observation suggests that maternal histones preferentially re-associate with daughter genomes near the location from which they originated—if old histones scattered randomly at replication, ancestral H3 retention patterns should more precisely anticorrelate with replication-independent turnover patterns, as is discussed in more detail below.

### Accumulation of Ancestral Histones at 5′ Ends of Genes

Why do old histone proteins accumulate near the 5′ ends of genes? We considered two alternative possibilities for classes of mechanisms causing this pattern. In the first mechanism, we reason that if histone proteins tend to maintain their locations along the genome, the 5′ enrichment of old histones implies that old 3′ histones are evicted and replaced by new histones during some phase of the cell cycle. However, previous measures of turnover in G1- or G2/M-arrested yeast [15],[26] cannot explain the 5′/3′ ratios we observe. Furthermore, we found that mutations in candidate 5′/3′-marking complexes such as cohesin [27],[28] or H3K4/K36 methylases [29] did not affect 5′-biased retention of old histones at target loci (Figure S8A).

A second possible explanation for widespread 5′ accumulation of ancestral histone proteins is that the histone proteins move from 3′ to 5′ over genes over time. This could result from RNA polymerase passage, because some RNA polymerases pass histone octamers in a retrograde direction during transcription [30],[31]. Although it is debatable whether this is true of Pol2 in vitro [32],[33], in vivo we previously observed that inactivation of Pol2 leads to a modest shift of nucleosomes from 5′ to 3′ [34], consistent with the idea that Pol2 movement normally shifts nucleosomes in a 5′ direction. To test whether this movement was related to transcription, we asked whether the 5′ peak of H3-HA accumulation shifted further 5′ with increasing transcription rate. We normalized all gene lengths to one, then plotted the HA/T7 ratio for all genes sorted by transcription rate (Figure S9). Consistent with the prediction of transcription-dependent retrograde movement, we did observe a subtle signal of H3-HA peaks shifting further 5′ at higher transcription rates. While this analysis could be confounded by the higher transcription rates seen over shorter genes, even when we focus on 1–2 kb genes, we observe that poorly transcribed genes exhibit a much flatter profile than genes expressed at average levels (Figure 2F), as expected if Pol2 transit were required for H3/H4 “passback.” Finally, we show below that per-gene estimates of passback exhibit significant correlation with Pol2 levels. Together, these results are most consistent with a model in which histone proteins move from 3′ to 5′ over coding regions over time (further detailed in the Discussion).

### Quantitative Estimation of Nucleosome Dynamics During Replication

**Figure 4:**
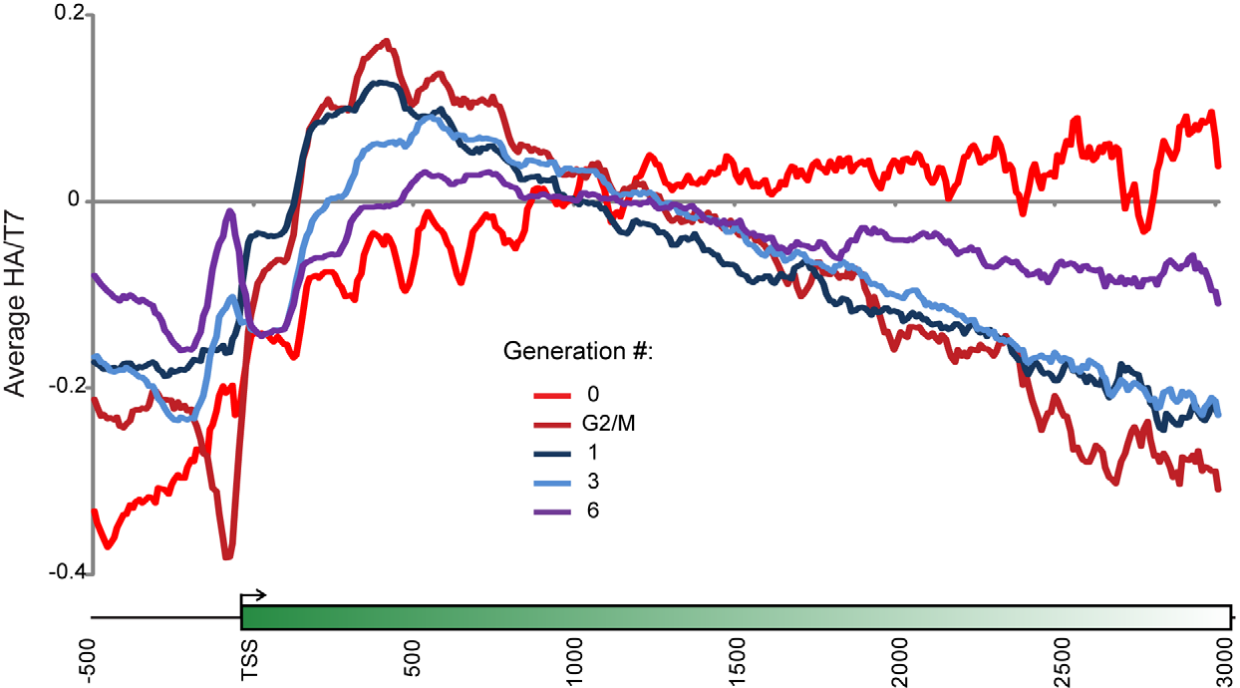
Kinetic analysis of ancestral H3 retention. HA/T7 ratios were measured genome-wide after recombination but before release (Gen 0), after release into nocodazole (G2/M), and after 1, 3, or 6 generations of growth post-release. Data for all genes were averaged and are plotted as indicated.

A key question we sought to address in this study is whether maternal histones re-associate near their original positions after passage of the replication fork. We reasoned that changes in HA/T7 patterns over the course of several generations might provide insight into the effects of replication on nucleosome dynamics. HA/T7 patterns change dramatically between arrest and 1 generation of release (with or without G2/M arrest) and then are very similar between 1 and 3 generations, before the background of nonswitching cells starts to dominate the profile at 6 generations (Figure 4). As expected, HA/T7 data at generation 0 exhibited widespread HA loss/T7 gain at promoters and +1 nucleosomes as a result of the rapid replication-independent turnover at these loci [13],[15],[17]. Importantly, to rule out the possibility that 5′ accumulation of H3-HA was an effect of our arrest-release protocol, we also measured HA/T7 distributions 6 h after inducing recombination in actively growing midlog cultures of yeast (Figures S3 and S10). Despite heterogeneity in switching times in this protocol (only 65% of yeast have switched from HA to T7 3 h after switch induction, 85% after 6 h), we nonetheless observed that HA/T7 distributions were remarkably similar in midlog-switching cells to HA/T7 patterns observed in cells undergoing the arrest/release protocol, with preferential ancestral histone accumulation at the 5′ ends of long, poorly transcribed genes.

**Figure 5:**
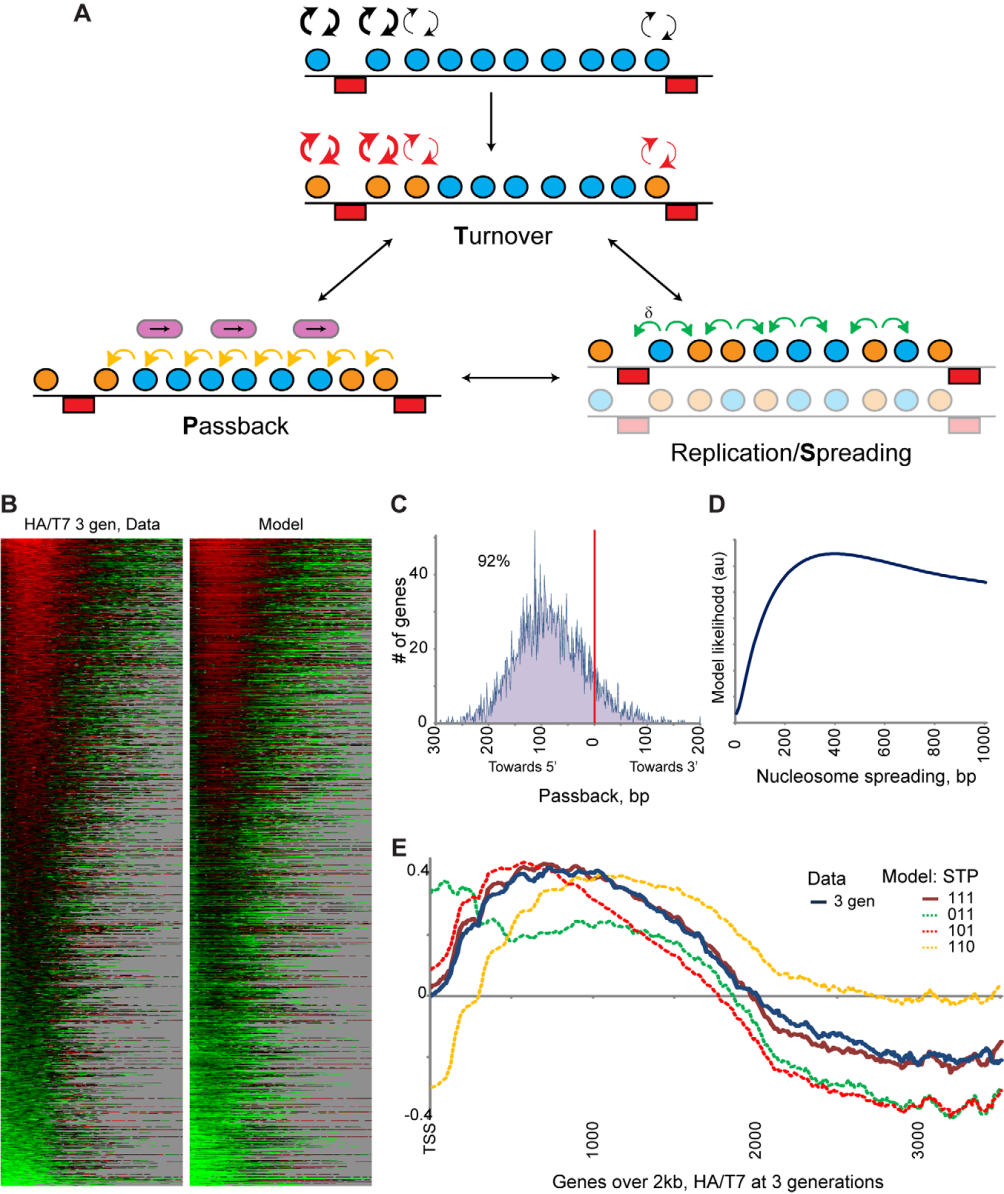
Quantitative modeling reveals three distinct dynamic processes. (A) Outline of quantitative model. From a given starting distribution, histones are subject to turnover [15], transcription-associated lateral movement (“passback”), and replication-mediated spreading. Model is described in detail in Text S1. (B) The model captures major features of the experimental data. HA/T7 ratios for experimental data and model predictions are shown for all genes as a heatmap. (C) Distribution of lateral passback parameter (per generation) for all genes. Note that the vast majority (92%) of genes were associated with retrograde 3′ to 5′ movement along coding regions. (D) Estimation of replication-based spreading of maternal histones. Model likelihood (Text S1) is plotted on the *y*-axis for various width spreading distributions (defined as 1 standard deviation of the Gaussian describing histone movement at replication—see Text S1 for model details). (E) Eliminating any of the three model features worsens fit to data. Plotted are averages at 3 generations for genes over 2 kb for data versus predictions of various models (“STP” refer to replication-mediated Spreading, replication-independent Turnover, and Passback). Note that the model eliminating turnover underestimates turnover effects, as histones that spread or are passed over the 5′ end of the gene are still eliminated in this model (i.e., in this model we effectively only eliminate turnover within CDS, not in intergenic regions), providing another basis for high loss of 5′ histones.

We asked whether these dynamic observations could be used to quantitatively rule out specific models concerning the mechanisms for segregation of maternal histones to daughter genomes. However, the resolution of this question is complicated by replication-independent processes we discuss above that can remove or shift ancestral histones, and that cannot be fully removed experimentally (for example, yeast will not proceed through the cell cycle in the absence of RNA polymerase). To understand the relationship between these issues, we designed an analytical model that accounts for three processes that affect H3 molecules in coding sequences (Figure 5A) and then examined the effect of removing any of the three. Briefly, our model includes a nucleosome-specific term for H3 turnover taken from prior experimental results [15], with H3 turnover resulting in loss of HA. In addition, it includes a gene-specific parameter accounting for lateral movement of histones (“passback”). Further, the model also includes a global parameter that describes the extent of histone “spreading” via dissociation/re-association during replication. Finally, the experimentally measured background of 2% nonswitching cells (Figure S1) was included. The free parameters of the model (describing global histone spreading and gene-specific lateral movement per generation) were estimated to maximize the likelihood of experimental observations (Text S1).

To account for any first-pass effects of Pol2 behavior during initial re-feeding of nutrient-depleted yeast (Figures S3 and S10), we examined this model with two starting conditions—the first started with a uniform genomic distribution of H3-HA, while the second started with the experimental distribution of HA/T7 observed after release into G2/M arrest (Figure 4). Both model variants predicted HA/T7 ratios with good correlations to the experimental data (Figure 5B shows data starting from a uniform distribution, Figure 5E and Figure S11 start from the G2/M distribution). Examination of estimated parameters revealed expected behaviors. For instance, the distribution of lateral histone movement estimates (Figure 5C) was strongly biased towards retrograde 3′ to 5′ movement of histones, consistent with the previously measured effects of *rpb1-1* inactivation on nucleosome positioning [34]. Passback values were also significantly (*p* = 9.6439e-19) correlated with transcription rate (Figure S12).

**Figure 6:**
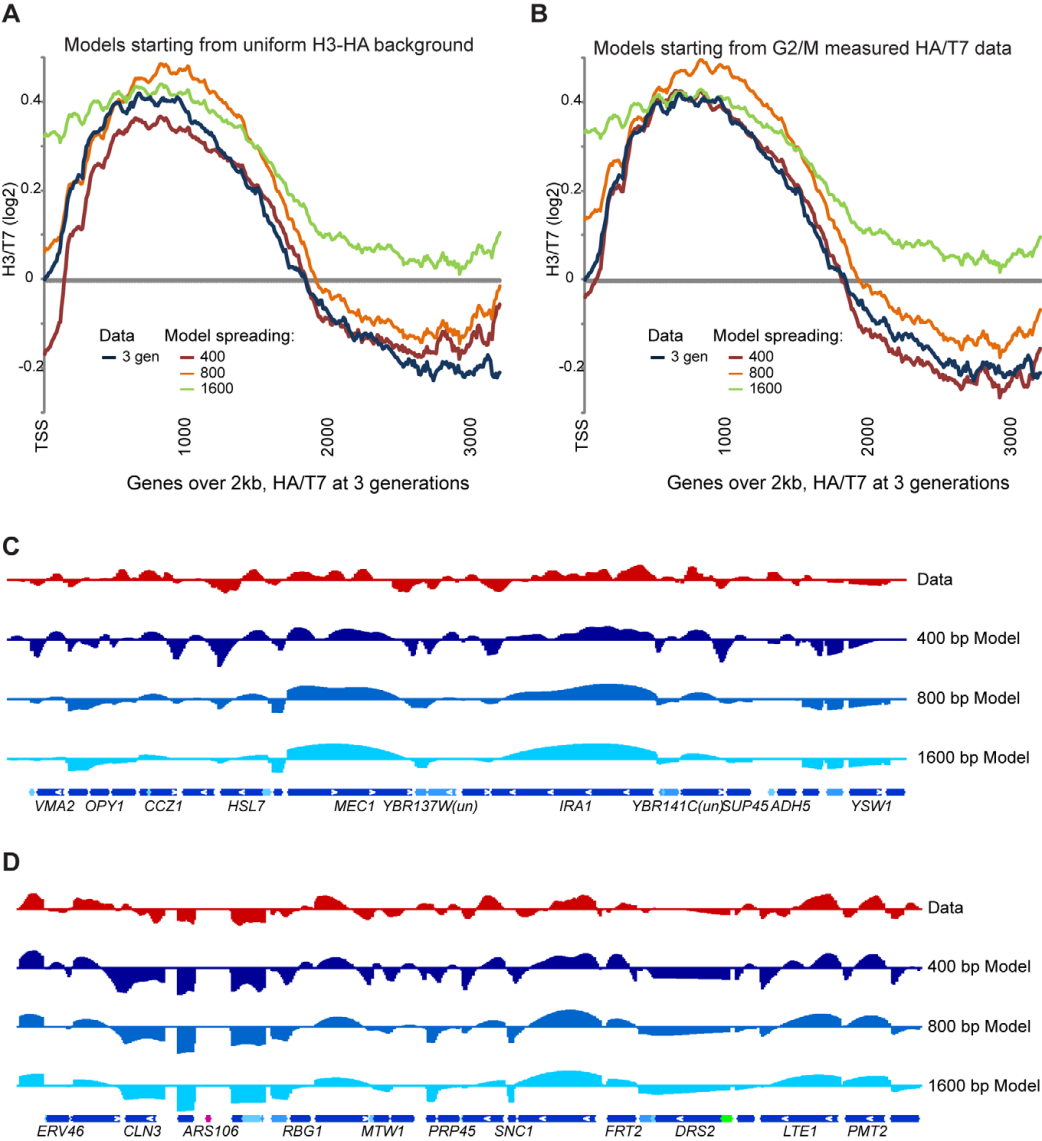
Dependency of histone dynamics model on spreading parameter. (A–B) Parameters in the quantitative model described in Figure 5 were re-optimized after fixing the spreading term to 400 bp (as in Figure 5), 800, or 1,600 bp. Data and simulations are shown averaged for genes over 2 kb for models starting with a uniform H3-HA distribution (A) or starting with the experimentally measured G2/M HA/T7 distribution (B). (C–D) Examples of data and three models with different spreading parameters. Genomic coordinates are chromosome 2 490–540 kb (C), and chromosome 1 60–110 kb (D). *Y*-axis shows measured (Data) or predicted HA/T7 values, in Log2.

Our model allows us to estimate the extent of histone movement during replication. Figure 5D shows the likelihood of the full model plotted for various values for replication-dependent histone spreading. The best fit model allowed histones to spread ∼400 bp in either direction, or roughly two nucleosome widths, during replication (more precisely, in this model two-thirds of histones stay within 400 bp of their original locations, as this value is the standard deviation of a Gaussian function describing spreading; see Text S1). Results from models with 800 and 1,600 bp spreading parameters are shown in Figure 6 for comparison. Our estimate of ±400 bp spreading is particularly interesting given electron microscopy results demonstrating that nucleosomes are destabilized over 650–1,100 bp around the replication fork on replicating SV40 minichromosomes [35],[36].

Elimination of any one or two of the three components of the model—spreading, turnover, or passback—resulted in significantly worse fits between model predictions and experimental data (Figure 5E). This can be intuited as follows. First, in the absence of histone spreading, unmitigated histone movement from 3′ to 5′ results in a much tighter 5′ ancestral histone peak and results in much more extensive change from one generation to the next than we observe. Second, eliminating histone turnover shifts the 5′ ancestral peak closer to the +1/+2 nucleosome. Third, preventing lateral histone movement results in a 3′-shifted, flatter ancestral histone profile.

While our model provided good quantitative fits of ancestral H3 patterns for many genes, we nonetheless note that many genes were not perfectly fit by this model. Generally, we found that the model poorly fit short genes, and overall the model almost universally predicted lower HA/T7 at the +N nucleosome (the last nucleosome in a gene) than was observed (Figures S11, S13). We ascribe these failures to the fact that we considered each gene in isolation and therefore did not model shifts of old nucleosomes from adjacent genes, which would result in poor fits over short genes in particular. Interestingly, the better fit at the +1 nucleosome than at the +N nucleosome is consistent with rapid promoter turnover more effectively isolating genes from one another at their 5′ ends in vivo.

Overall, the strong correlation between our model and the experimental data supports the hypothesis that at least three dynamic processes affect nucleosomes and shape the landscape of ancestral histone retention and provide the first quantitative estimate of maternal histone dynamics during replication.

### Topoisomerase I and the H4 N-Terminal Tail Play Roles in Establishing the 5′/3′ Gradient of Ancestral H3 Molecules

**Figure 7:**
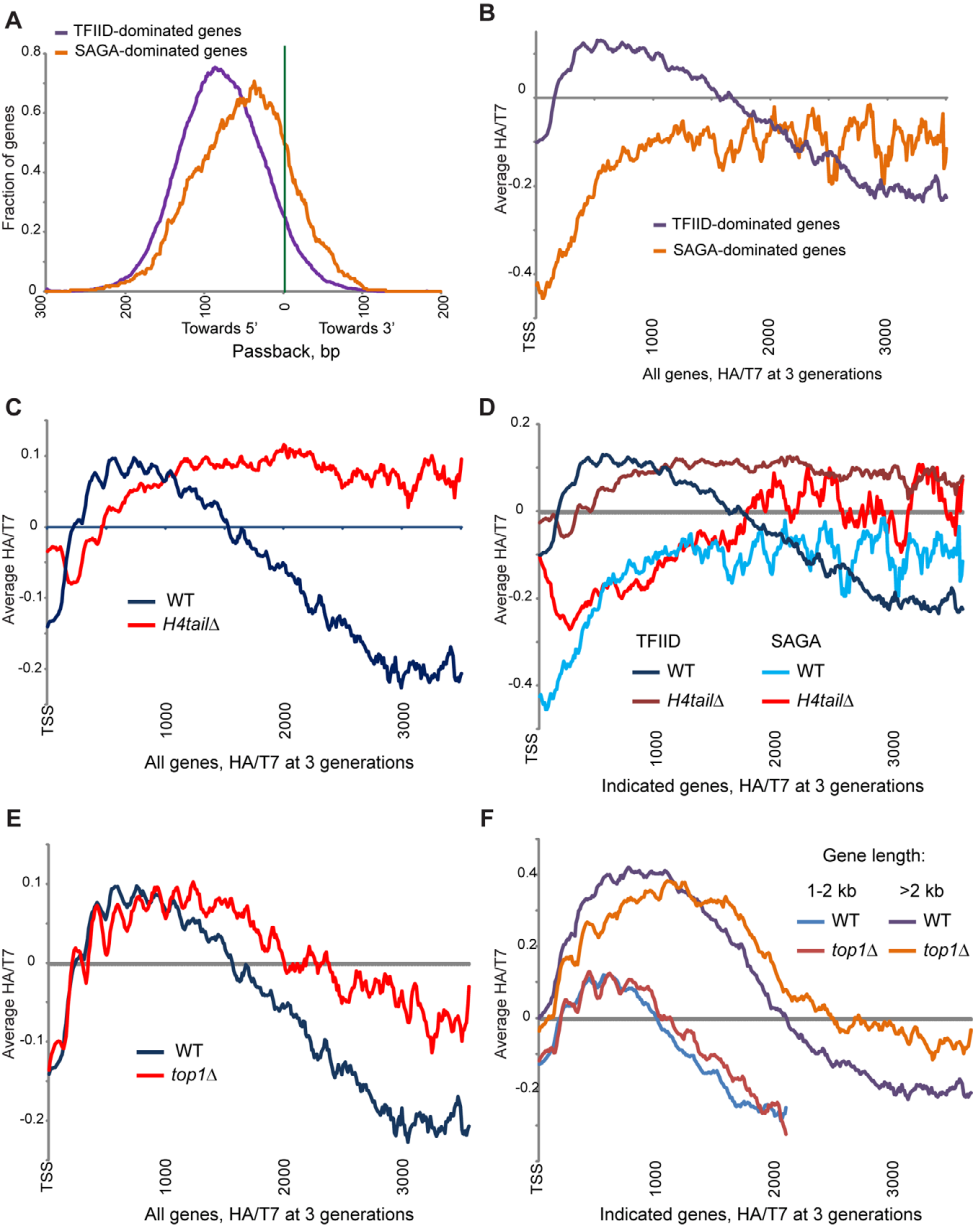
Mutants affecting ancestral histone retention. (A) Distribution of lateral nucleosome distances from model (Figure 5). Shown are the passback parameters for SAGA-dominated and TFIID-dominated genes as defined in Huisinga et al. [38]. (B) TFIID-dominated genes preferentially accumulate 5′ H3-HA. Averages of 3 generation experimental data are shown for the indicated gene classes. (C) H4 tail deletion dramatically reshapes the landscape of ancestral histone retention. Yeast carrying an N-terminal H4 tail deletion were processed as in Figure 1A–B, and averages for all genes are plotted as indicated. We note that this strain has retained a wild-type *HHT2-HHF2* locus for viability, so results must be interpreted with caution. However, we find similar but less dramatic effects in an H4K5,12R mutant (Figure S8B), supporting the observation here that passback is affected by the H4 N-terminal tail. (D) H4 tail deletion preferentially affects TFIID-dominated genes. Data for wild-type and *H4tailΔ* yeast are plotted for the indicated gene classes. (E) Topoisomerase I plays a role in 5′ accumulation of ancestral histones. *top1Δ* yeast were processed as in Figure 1A–B, and averages for all genes are plotted as indicated. (F) *TOP1* deletion affects 5′ passback preferentially at long genes. Data for wild-type and *top1Δ* yeast are plotted for the indicated gene classes.

To further investigate the mechanism of 5′ accumulation, we asked whether gene-specific passback parameters were correlated with specific gene annotations (Table S2) [34],[37]. Interestingly, we find that the estimated passback distance was much greater at TFIID-dominated (“growth”) genes than at SAGA-dominated (“stress”) genes (Figure 7A) [38]. As a result, 5′ accumulation was much more pronounced at TFIID-dominated than at SAGA-dominated genes (Figure 7B). Almost every described aspect of chromatin structure and gene expression, from nucleosome positioning to evolutionary lability (reviewed in [39],[40],[41]), differs between these two broad types of genes. Mechanistically, one interesting correlate is that TFIID recruitment has been proposed to be mediated in part by acetylation of the N-terminal tail of histone H4 [38],[42].

To investigate this link experimentally, we examined whether mutations of the H4 tail influenced ancestral histone H3 retention. In an H4K5,12R mutant that cannot be acetylated on these two tail residues, the 5′-biased HA/T7 was partially lost (Figure S8B), consistent with the possibility that acetylation of H4 tail lysines may contribute to H3/H4 passback. We also deleted the H4 N-terminal tail, although in this strain background this mutation proved lethal and so all recovered strains retained a wild-type copy of the H4-H3 locus (*HHF2-HHT2*). Thus, results with this strain must be interpreted with extreme caution, as we do not know the effect of wild-type, untagged nucleosomes on the behavior of the epitope-tagged histones.

Nonetheless we present here results of mapping of HA and T7 3 generations after release from the HA/T7 tag swap, since the H4 tail deletion has dramatic effects on global nucleosome dynamics (Figure 7C), with low HA/T7 at 5′ ends followed by a nearly flat profile over the remainder of coding regions. This profile suggests a requirement for the H4 tail in H3/H4 passback, and possibly on replication-mediated spreading (see Figure 5E). Interestingly, the effect of H4 tail deletion was much more pronounced at TFIID-dominated genes (Figure 7D), suggesting that the exaggerated H3/H4 passback inferred at these genes involves the H4 tail. The effects of the H4 tail deletion were not simply due to the extensive changes in the transcriptome [43], as we measured changes in genome-wide RNA Pol2 localization in our H4 mutant strains, finding that the relationship between Pol2 levels and HA/T7 behavior qualitatively changed in this mutant (Figure S14). While we must be cautious interpreting results obtained with the H4 tail deletion, the fact that H4K5,12R mutants (which were viable and did not retain any wild-type H3/H4) also exhibit diminished 5′ bias in ancestral H3 retention provides independent support for a key role for the H4 tail in H3/H4 passback.

We also explored the role of supercoiling in the 5′-biased retention of old histones. Topoisomerases relax DNA supercoiling and thereby help to maintain chromatin architecture. Transcription of DNA templates by Pol2 differentially affects supercoiling in front of and behind the passing polymerase, thereby differentially affecting 5′ and 3′ nucleosomes [44],[45]. To assess the role of this activity in 5′ accumulation of old histones, we examined the consequences of inactivation of the major topoisomerase Top1, which in vitro can resolve both negative and positive supercoils [46],[47]. Cells lacking Top1 showed reduced 5′ bias in ancestral nucleosome accumulation (Figure 7E), indicating that resolving DNA topology problems before or after passage of the transcription or replication machinery influences the mobility and/or stability of nucleosomes. Consistent with expectations of a greater buildup of supercoils over longer transcription units, we confirmed a stronger effect of *TOP1* deletion at longer genes (Figure 7F).

### Replication Timing, Chaperones, and Ancestral Histone Retention

We finally turn to the role of replication factors in ancestral histone retention. We first asked whether replication timing affected H3-HA retention. Nucleosomes surrounding early-firing origins tended to lose H3-HA more rapidly than late-firing origins (unpublished data), but this likely stems from the fact that replication timing correlates with replication-independent turnover [23],[26]. Focusing only on nearby coding regions (Figure S15), we found that late-replicating genes were associated with slightly 5′ shifted ancestral H3 peaks relative to genes near early origins (consistent with decreased spreading or turnover), suggesting that different replication forks might affect chromatin in different ways, although the modest effect precludes a stronger interpretation.

**Figure 8:**
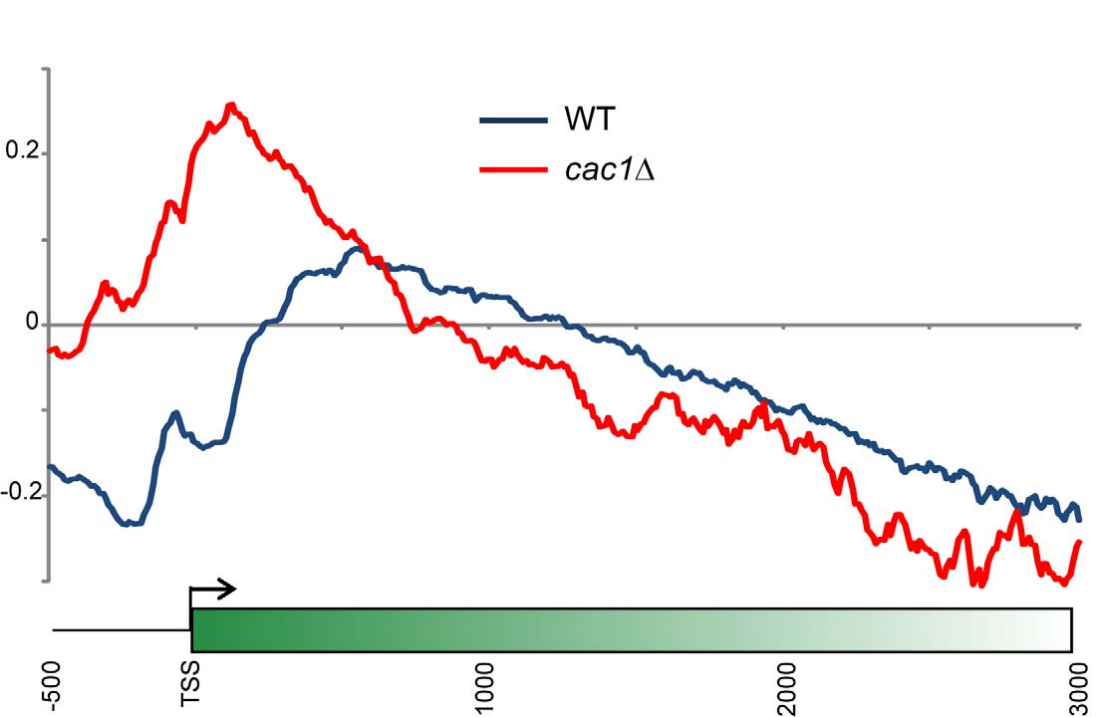
Effects of Chromatin Assembly Factor-1 complex on ancestral H3 patterns. Yeast lacking CAF-1 subunit Cac1 were processed as in Figure 1A, and HA/T7 ratio averages are shown for all genes in wild-type and *cac1Δ* mutants 3 generations after release.

To directly address the role of fork-associated chromatin proteins in histone spreading at replication, we examined mutations of PCNA and Chromatin Assembly Factor (CAF-1), which plays a key role in replication-coupled histone deposition [48],[49]. Three different mutants of PCNA that disrupt interactions with replication proteins or with replication-coupled chromatin-assembly factors showed only minor effects on 5′ retention of ancestral H3 at target genes *SPA2* and *BUD3* (Figure S8C). In contrast, ancestral H3 retention at the 5′ ends of these target genes was slightly *increased* upon deletion of the CAF-1 subunit *CAC1* (unpublished data). To further explore the role of CAF-1 in histone retention patterns, we deep sequenced HA and T7 tags from *cac1Δ* yeast 3 generations after release (Figure 8). These data show a dramatic 5′ shift in the peak of ancestral H3 retention in these mutants. This shift is most consistent with a decrease in histone turnover at the 5′ ends of genes in this mutant, which we have independently confirmed using G1-arrested yeast expressing p*GAL*-driven Flag-H3 [50]. However, we cannot rule out the possibility that the role of CAF-1 in retention of old histones in 5′ and promoter regions involves interactions with PCNA during DNA replication. Interestingly, the 5′ accumulation observed in wild-type yeast is otherwise little changed in the CAF-1 mutant over long genes (Figure S16), suggesting that 3′ to 5′ movement of histones is normal, and that preferential retention of old histones at their maternal locations may be carried out by alternative histone chaperones such as the Hir complex or Asf1 in this mutant. Unfortunately, both *hir* and *asf1* mutants are lethal in our strain background (likely because our strain carries only one copy of the H3/H4 gene pair [51]), preventing us from testing this hypothesis.

### Consequences for Histone Modification Patterns

Our results are most consistent with histone retrogression from 3′ to 5′ over genes, which raises the question of whether old histones carry modifications associated with mid- and 3′ coding regions (e.g., H3K36 and H3K79 methylation) towards the 5′ end of genes. Alternatively, there could be active erasure of these modifications. We therefore compared genes exhibiting high levels of ancestral H3 retention with prior genome-wide analyses of histone modifications [52],[53]. Histone modification patterns generally conformed to the patterns expected based on transcriptional behavior—genes that retain high levels of ancestral histones are poorly transcribed (Figure 2D), and correspondingly exhibit low levels of transcription-related marks H3K9ac, H3K14ac, H4ac, and H3K4 methylation (Figure S17 and unpublished data). However, these are all 5′-biased marks [19],[29],[52],[54], and based on retrograde movement of old histones are therefore not expected to accumulate with age.

**Figure 9:**
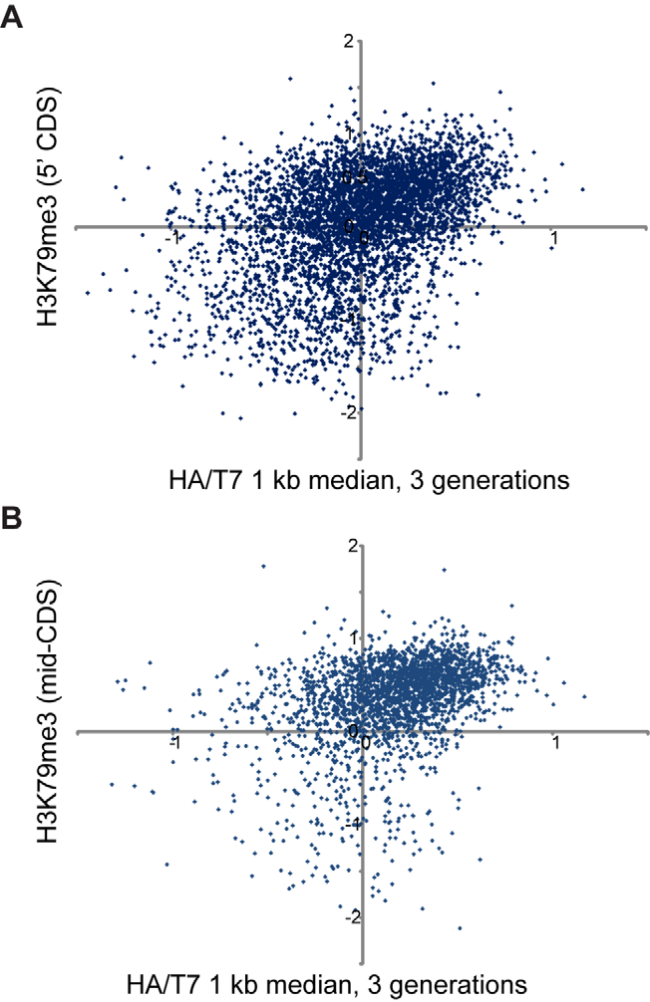
Ancestral H3 retention and histone modification patterns. (A) Scatterplot of previously measured H3K79me3 [52] levels averaged over the 5′ CDS of genes versus the median HA/T7 for the 5′ 1 kb of each gene. (B) As in (A), for K79me3 averaged over mid-CDS of each gene.

More interestingly, we found that genes with high levels of old nucleosomes were enriched for H3K79me3 throughout their coding regions, particularly at the 5′ end (Figure 9, Figure S17). The H3K79 methylase is nonprocessive, indicating that K79 methylation status should essentially act as a timer [55]. Further, analyses of genome-wide H3K79me3 patterns show anticorrelation between this modification and locations of high nucleosome turnover [15],[56], supporting the idea that K79me3 identifies old H3 protein. We recently confirmed that old H3 protein is enriched for H3K79me3 by mass spec analysis of old nucleosomes (D. DeVos, FvL et al., submitted).

Finally, we also observed higher levels of H3K36me3 at 5′ and mid-CDS of genes exhibiting elevated ancestral histone retention relative to genes with intermediate H3-HA retention (Figure S17). This observation is consistent with the above hypotheses that old histones move from 3′ to 5′ and thus might carry typical mid-CDS and 3′-end histone modifications to the 5′ ends of genes (Figure S18). Together, these results provide further evidence that our system accurately captures the behavior of old histones.

## Discussion

The fate of stable proteins in rapidly dividing cells is of great interest for fields from protein damage to aging to epigenetic inheritance. In particular, models for the inheritance of chromatin-based information [5]–[7],[12],[57],[58] require a quantitative understanding of the fate of specific maternal histone proteins during the disruptive replication process. Some models for epigenetic inheritance of chromatin states require that old nucleosomes are retained near their original positions, whereas other models (such as those based on replication timing; [59]) are less sensitive to the fates of old histones. However, due to the lack of methods to directly track histone dispersal during replication, these models have not been experimentally tested in vivo. Here, by using a novel genetic pulse-chase assay, we characterize ancestral histone retention patterns across the yeast genome. By accounting for known replication-independent processes, we used these data to estimate the effects of replication on histone movement, finding that H3/H4 are retained close to their original locations during replication. We also identified a number of mutants that affect various aspects of ancestral H3/H4 movement and retention.

### Ancestral Histones Accumulate at the 5′ Ends of Genes

Most surprising to us was the observation that ancestral H3 molecules accumulate near the 5′ ends of coding regions, peaking around the +3 nucleosome. The high HA/T7 ratio observed at the 5′ ends of genes is not an artifact of the epitope tags used, as we have observed the converse behavior (high T7/HA) when we switch the epitope tags used (unpublished data). Furthermore, this unusual behavior is not an artifact of the conditions used for growth arrest and release, as we observe a similar 5′/3′ gradient of H3-HA when yeast are subjected to the epitope switch during active midlog growth (Figure S10).

What is the mechanistic basis for the 5′/3′ gradient of HA/T7 we observe? We consider two classes of mechanisms—in one, histone proteins do not move laterally and the 5′/3′ gradient results from preferential loss of 3′ H3/H4, while in the other the gradient results from lateral histone movement combined with loss at the 5′ end. While we cannot definitively answer which mechanism explains our results, we strongly disfavor a model with preferential 3′ nucleosome eviction and no lateral movement based on the following observations. First, we tested a number of relevant mutants for changes in the 5′/3′ HA/T7 bias (Figure S8). Loss of H3K4 methylation (a 5′-biased histone mark) or H3K36 methylation (a mid and 3′-biased histone mark) did not affect HA/T7 patterns at selected target genes. Similarly, 5′ retention of ancestral H3 was unaffected by mutants of cohesin, whose loading is associated with regions of high H3/H4 turnover and which accumulates at the 3′ ends of genes [27],[28]. Second, direct measurements of H3/H4 turnover using a p*GAL*-driven epitope tagged H3 do not provide evidence for ubiquitous 3′ histone replacement during G1 arrest [15], during G2/M arrest [26], or in unsynchronized yeast [15]. Thus, while we cannot definitively rule out some cryptic 3′ replacement event in this system, all direct tests have failed to support this hypothesis.

Conversely, multiple observations support the hypothesis that H3/H4 proteins move from 3′ to 5′ over protein-coding regions over time. First, seminal in vitro studies on transcription of nucleosomal templates showed that several RNA polymerases can transcribe through a nucleosome without displacing the H3/H4 tetramer. The proposed mechanism by which histones remain associated with the DNA is a “bubble propagation” mechanism—DNA partially unwraps from the histones, RNA polymerase enters, and DNA behind the polymerase re-associates with the histone octamer, resulting in a net retrograde movement of histones after the polymerase has passed. This mechanism is relatively well established for SP6 polymerase and RNA Polymerase III [30],[31], whereas there is some controversy regarding the effect of RNA Polymerase II on nucleosome positioning [32],[33]. Of course, it is not unreasonable to expect that nucleosome movement during transcription in vivo will also be affected by polymerase-associated factors such as histone chaperones and ATP-dependent remodelers that are not present in the in vitro systems. In any case, these studies provide a plausible mechanism by which RNA polymerase transit results in retrograde nucleosome movement.

Second, we have previously found that inactivation of Pol2 using the temperature-sensitive *rpb1-1* allele results in a net 5′ to 3′ shift in the majority of coding region nucleosomes [34], consistent with the hypothesis that polymerase transit normally shuttles nucleosomes from 3′ to 5′. Third, highly transcribed genes (such as those encoding ribosomal proteins) in yeast paradoxically exhibit very tightly spaced coding region nucleosomes (e.g., 155–160 bp between adjacent nucleosomes rather than ∼165 bp), and this tight spacing relaxes upon Pol2 inactivation, again consistent with nucleosomes being passed upstream during transcription [34]. Taken together with the absence of any evidence for 3′ H3/H4 eviction, we therefore argue that the most parsimonious explanation of the surprising 5′ accumulation of ancestral histones is retrograde movement of histones over genes against the direction of transcription. Note that while we favor the hypothesis that the act of RNA polymerase transit itself is the mechanism linking transcription to H3/H4 passback, polymerase is not the only candidate factor leading to retrograde histone movement. Notably, we found that *top1Δ* mutants exhibit diminished signatures of H3/H4 passback (Figure 7), and this decrease was stronger at longer genes, suggesting the possibility that some aspect of cleavage and rotation of twisted DNA by Top1 contributes to the passback observed. However, it is also possible that Top1 differentially affects histone turnover in 5′ and 3′ regions or affects passback by affecting Pol2 passage [60].

We analytically assess several predictions of the “passback” model. First of all, if RNA polymerase transit were the driver of retrograde histone movement, then one might predict that passback should correlate with transcription rate. We find the expected correlation to be statistically significant (*p* = 9.6439e-19, Figure S12) but weak nonetheless (R = 0.12). Importantly, we previously observed that 5′ to 3′ nucleosome movement in *rpb1-1* mutants was also significantly but poorly correlated with transcription rate [34]. The reason for the mediocre correlation between polymerase abundance and passback is hinted at by the fact that TFIID-dominated genes exhibit much greater passback values than do SAGA-dominated genes (Figure 7A). We have previously noted that SAGA-dominated (“stress”) genes exhibit higher levels of H3 turnover, *per polymerase*, than do TFIID-dominated genes [15]. In vitro, a single polymerase's transit displaces an H2A/H2B dimer from the histone octamer, but a second polymerase encountering a histone hexamer will displace the remaining histones [61],[62]. Coupled with the observation that SAGA-dominated genes exhibit larger “bursts” of polymerase, this suggests that closely spaced polymerases are required for H3/H4 eviction over coding regions, but evenly spaced polymerases leave time for dimer replacement on damaged nucleosomes [54],[62]. We believe this model also explains some of the behavior of ancestral histones in this study—SAGA-dominated genes display little passback and overall diminished levels of ancestral H3 (Figure 7A–B), an expected consequence of the loss of old histones via turnover. Correlations between polymerase and passback are therefore expected to be subtle—at increasing transcription rates, we expect an increased likelihood of a closely spaced pair of polymerases, and the resulting H3/H4 eviction would eliminate any trace of the passback that had occurred to that point.

It is important to note that the transcription-dependent passback postulated here cannot simply be interpreted as a model in which every round of polymerase passage shifts the histone octamer upstream by one position (∼165 bp). In Figure 7A, our estimates of passback per cell cycle have a mean of ∼90 bp at TFIID-dominated genes, less than the spacing between adjacent nucleosomes. If taken literally, these values would be difficult to reconcile with the observation that the majority of yeast nucleosomes are well positioned [20]. Instead, we interpret the passback values in terms of probability that an octamer will be passed back in a given cell cycle in each cell—a passback value of ∼80 bp suggests that there is a 50% chance that histones on a given gene will be shifted back one position towards the 5′ end in a single cell cycle. Physically, we imagine that polymerase passage results in relatively short retrograde movement of H3/H4, which then have some probability of returning to their original position, and some probability of shifting to a new upstream location.

Our results show a surprising pattern of ancestral histone retention in yeast, with old histone proteins accumulating near the 5′ ends of genes—the histone proteins located at the +3 nucleosome are the oldest histone proteins over a typical yeast gene. These data are best explained by a model in which H3/H4 proteins shuttle from 3′ to 5′ over coding regions over time, with eventual loss of old histone proteins when they are eventually moved into the +1 and +2 nucleosome positions.

### Maternal Histone Spreading During Replication

The process of genomic replication is enormously disruptive to chromatin structure, as the melting of the DNA double helix is accompanied by histone dissociation from the genome [4]–[7]. Thus, understanding where maternal histones re-associate relative to the locus from which they were evicted is a key constraint for understanding the potential of chromatin as an epigenetic information carrier. The ideal experiment for measuring this would be to epitope tag the histones at one specific locus (e.g., the +5 nucleosome over *BUD3*) in a large population of yeast, allow replication to proceed, and measure the new locations of the tagged histones. Despite numerous attempts, this type of tagging has proven technically intractable to date. Here we measure instead the bulk distribution of ancestral histones. Importantly, this still provides information on locus-specific histone behavior—as turnover rates are not homogeneous across the genome, even before we release yeast into the cell cycle the landscape of H3-HA exhibits variability (Figure 4, see generation 0), and so in effect only a subset of ancestral locations are epitope-tagged before release. This enables us to infer the dynamic behavior of histone proteins during replication via analysis of the evolution of the H3-HA distributions over time.

Two observations provide an intuition regarding the effects of replication on histone locations. First, ancestral histone retention exhibits the expected anticorrelation with replication-independent turnover (Figure 3A). However, old histones are more efficiently retained at cold (low turnover) loci that occur in long cold domains, whereas short domains of cold nucleosomes lose ancestral histones over time. This observation is inconsistent with two extreme models for histone behavior during replication—if old histones were to completely dissociate from the genome during replication and randomly re-associate with the genome, then ancestral histone retention should precisely recapitulate turnover measurements. Conversely, if old histones were to reassociate precisely with their original locations, then ancestral retention should essentially integrate turnover for multiple generations. Thus, some process that shuffles histone proteins locally must be invoked along with turnover to shape the ancestral retention landscape.

In principle, the preferential retention of old histones on longer genes could simply result from passback—shorter genes will more quickly have all of their histones passed “over a cliff” at the 5′ end. However, we find relatively static 5′/3′ gradients of old histone retention over time (Figure 4). While it is the case that H3-HA domains gradually shorten over time as predicted by the model that passback results in old histones being moved to promoters where they are replaced (unpublished data), this effect is subtle and is quantitatively much less dramatic than predicted from passback alone. This leads to the second intuition regarding histone spreading during replication. Many examples exist for relatively static gradients in biological systems being established via a combination of directional active transport coupled with passive diffusion. Most relevant in our opinion is the “pump leak” model [63] for membrane ion gradients—active transport of ions across membranes, coupled with a passive leak of ions back into the cell, results in a static gradient. Here, we envision transcription-related passback as the active transport mechanism, with spreading during replication being somewhat analogous to the leak that results in a steady gradient rather than a continuous 3′ to 5′ march of histone proteins.

We present a quantitative model that recapitulates our experimental data with only three dynamic processes—turnover, passback, and spreading. Locus-specific turnover rates were previously measured [15] and are not fit by the model. Passback is estimated for each gene separately, while spreading is a single global parameter affecting all histones. Thus, our model has 4,811 free parameters, which are used to fit over 100,000 HA/T7 ratios. This model does not overfit the data, and this can best be appreciated by the fact that eliminating a single parameter (spreading) greatly diminishes the agreement between model and data.

Using this model, we estimate that maternal histones spread little (∼1–2 nucleosomes) during replication. This value has not been measured before but is consistent with several related observations. First, electron microscopy studies on replicating chromatin show a stretch of ∼650–1,100 bp of nucleosome-free DNA surrounding replication forks [35],[36], consistent with histone movement of ±400 bp we estimate here. Second, histone proteins are retained in cis during in vitro replication even in the presence of competitor DNA [64]–[67], indicating that histones do not freely diffuse away from replication forks but likely are retained locally. Finally, we previously observed that upon gene repression, loss of the active chromatin mark H3K4me3 occurs during S phase, but at very highly methylated nucleosomes H3K4me3 does not return to baseline levels immediately, with methylation levels falling little more than the 2-fold predicted by a dilution-based mechanism (see Figure S5 in [68]). This final result indicates that “overmethylated” old histone proteins are retained near their original location, since extensive spreading of old histone proteins would enable a greater than 2-fold drop in methylation levels during S phase.

Together, these results support the prospect of chromatin as a “sloppy” epigenetic information carrier (“sloppy” in the sense that some spreading of histones will preclude mononucleosome-resolution information passage) [69], even if chromatin-based inheritance occurs infrequently [1]. Thus, chromatin states are unlikely to be inherited with mononucleosome precision, a view consistent with the fact that most or all proposed epigenetic chromatin domains are associated with long (>1 kb) blocks of histone modifications such as H3K9me3 or deacetylated H4K16 (reviewed in [54]).

### Mutant Studies

To further investigate the mechanisms underlying the patterns of ancestral H3 retention, we assessed HA/T7 ratios at target genes in 12 mutants and further characterized HA/T7 genome wide for three of these mutants. Interestingly, a number of histone modifying factors, including Swd1, Swd3, Rtt109, Nhp6, and Set2, had either no effect or subtle effects (e.g., Rtt109) on the 5′ accumulation of old histones at our target genes (Figure S8). These results suggest either that these mutants will have subtle global effects on ancestral H3 retention or that they have more localized roles that do not extend to the two target genes on which we focused.

The three mutants we characterized at full genome coverage each had a distinct effect on ancestral H3 retention. Most dramatically, loss of the H4 N-terminal tail abolished the 5′ accumulation of ancestral histones—while the H4 tail deletion results are complicated by the retention of wild-type H3/H4 in this strain, the fact that similar results were obtained with clean H4K5,12R mutants (Figure S8B) provides independent support for observations obtained with the H4 tail deletion. The mechanistic basis for the loss of 5′ H3-HA retention is unknown to us—a flat HA/T7 profile is of course consistent with complete loss of passback. Alternatively, the observed profile in this mutant could be consistent with complete shuffling of maternal histones every generation, which as described above would be expected to more closely recapitulate a turnover-dominated profile. Importantly, loss of the H4 tail also affects H3/H4 turnover—in Figure 7D, the increased HA/T7 ratio at the 5′ ends of SAGA-dominated genes suggests a decrease in histone turnover in this mutant, and we have independently confirmed a decrease in replication-independent turnover in this mutant (F.v.L., manuscript in preparation). Analysis of Pol2 ChIP in H4 tail deletions shows that the effects of the H4 tail do not simply reflect altered transcription but instead reflect a change in the relationship between RNA Polymerase and histone dynamics over genes in this mutant (Figure S14).

We also observe a similar, albeit muted, effect of Topoisomerase I on the 5′ accumulation of ancestral histones. Interestingly, loss of both topoisomerase I and II affects nucleosome occupancy and dynamics in *S. pombe*, indicating that topoisomerases play key roles in histone dynamics [60]. Here, we find that *top1Δ* mutants exhibit diminished 5′ accumulation of ancestral H3 and that this effect is stronger at longer genes than at shorter genes. As RNA polymerase passage will cause greater changes in supercoiling over longer genes, the preferential effects of Top1 on longer genes is consistent with the observation in *S. pombe* that topoisomerase mutants show evidence of stalled or slowed RNA polymerase over longer genes [60]. In addition to its role in transcription, topoisomerase I plays a key role in replication [45]. We note that the profile of *top1Δ* mutants here most closely mimics the predictions of our analytical model with both passback and spreading being compromised, but since neither of these is likely to be completely eliminated in *top1Δ* mutants, more detailed kinetic analyses will be required to make a quantitative statement about the role of Top1 in replication-related movement of histones.

Finally, we assessed the role of the histone chaperone CAF-1 in ancestral H3 retention. To our surprise, we found that H3-HA exhibited even stronger 5′ accumulation in this mutant, with the 5′ peak of HA/T7 occurring closer to the +1 or +2 nucleosome (compared to the +3 peak location for wild-type strains). This result most closely matches the predictions of a model in which H3/H4 turnover has been slowed without loss of passback or spreading (Figure 5E). We recently tested this prediction using an alternative system for measuring replication-independent turnover (p*GAL*-driven Flag-H3) and confirmed the prediction that *caf* mutants affect replication-independent histone replacement [50]. As CAF-1 and the Hir complex are known to complement one another in yeast, we predict that a *caf hir* double mutant would be necessary to uncover effects of replication-coupled spreading. Unfortunately, since both *hir* and *asf* mutants are lethal in our strain background, this prediction cannot be tested at present.

### Perspective

Taken together, our results provide a surprising view of histone dynamics over multiple generations, with 5′ accumulation of ancestral histone proteins over coding regions and little evidence for preferential histone retention at epigenetically regulated loci such as subtelomeric genes. One unanticipated implication of this observation is that 3′ histone marks are expected to move towards the 5′ ends of genes over time, thereby shaping histone modification profiles (as we document in Figure 9 and Figure S17). This potentially necessitates mechanisms for erasure of these inappropriate marks in order to maintain accurate encoding of gene polarity. However, we note that active erasure of H3K4me3 after gene repression occurs most efficiently at 5′ ends of genes, whereas nucleosomes over coding regions mostly lose H3K4me3 by passive dilution ([68], see Figure S9). If other old histone marks are not erased over coding regions, then we speculate that the accumulation of old histone proteins at the +3 nucleosome could potentially provide a mechanism by which a gene's transcriptional history could be integrated to play a role in regulation of the transition from transcriptional initiation to elongation.

Most importantly, we find that old histones do not re-associate with daughter genomes at precisely the locus from which they dissociated. Thus, any inheritance of chromatin states must occur at the scale of ∼5–10 nucleosome domains rather than at single nucleosome resolution. These results therefore constrain the maximum amount of information theoretically carried by chromatin between generations. It will be of great interest in future studies to identify mutants that affect histone movement during replication and to measure their effects on the stability of epigenetic inheritance and to measure how maternal histone incorporation differs between leading and lagging strand daughter genomes.

## Materials and Methods

### Yeast Strains and Growth Conditions

For tag switch experiments, yeast cells were grown overnight in YPD in the presence of Hygromycin B (200 µg/mL, Invitrogen). The cells were then diluted 1∶10 into fresh YPD and incubated for 30–36 h. Recombination was induced by the addition of 1 µM β-estradiol (E-8875, Sigma-Aldrich). Subsequently, cells were diluted 1∶25 in fresh YPD media to release the cells back into the cell cycle and kept in log phase by 1∶2 dilutions into fresh media after each population doubling. Samples were taken after 1, 2, 3, and 6 cell divisions or after 5 h of G2/M arrest. The number of population doublings was determined by microscopy and OD. G2/M arrest was induced by addition of 15 µg/ml Nocodazole (Sigma-Aldrich) and confirmed by FACS analysis. Strains are listed in Table S3. Gene deletion mutants isogenic to strains NKI2048, NKI2148, and NKI2048 were made by homologous recombination using KanMX and/or NatMX selection markers. Gene deletion mutants isogenic to NKI4128 were made by crossing NKI4114 with gene deletion mutants from the MAT**a** yeast knock-out collection using Synthetic Genetic Array methods. Histone mutants were made by transformation of strain NKI2148 with a *HHF2-HHT2* CEN plasmid (pMP9), subsequent deletion of the tagged *HHF2-HHT2* locus, followed by transformation with a PCR fragment encoding wild-type or mutated *HHF2* in combination with tagged *HHT2*. Deletion of the wild-type locus was confirmed in H4K5,12R mutants, whereas all surviving H4 tail deletion mutants retained a copy of the wild-type *HHT2-HHF2* locus.

### Chromatin Immunoprecipitation (ChIP)

ChIP was performed as described previously [13],[70] with the following modifications. All steps were done at 4°C unless otherwise indicated. Following cell lysis by bead beating the insoluble chromatin of 1×10^9^ cells was washed, resuspended in 400 µl FA lysis buffer (50 mM HEPES-KOH [pH 7.6], 150 mM NaCl, 1 mM EDTA, 1% Triton X-100, 0.1% sodium deoxycholate), and sheared using a Bioruptor (Diagenode) for 6 min with 30 s intervals at high. The soluble fraction was diluted 3-fold in buffer 15 mM Tris-HCl pH 7.4, 50 mM NaCl, 1.5 mM CaCl_2_, 5 mM β-meracptoethanol, 5 mM MgCl_2_, after which 25 units of micrococcal nuclease (Worthington) were added. The digestion reaction was incubated 20 min at 37°C and stopped by the addition of 10 mM EDTA and 10 mM EGTA; tubes were placed on ice. The majority of obtained fragments was around 150 bp, as determined on a 2% TAE agarose gel stained with ethidium bromide. The isolated chromatin of the equivalent of 3×10^8^ cells was immunoprecipitated overnight at 4°C using magnetic Dynabeads (Invitrogen), which were previously incubated with antibody O/N at 4°C.

### Real-Time PCR

ChIP DNA was quantified by real-time quantitative PCR using the SYBR Green PCR Master Mix (Applied Biosystems) and the ABI PRISM 7500. An input sample was used to make a standard curve, which was then used to calculate the IP samples, all performed in the 7500 fast system software. Primers used for qPCR are listed in Table S4.

#### Linear amplification of DNA

The samples were amplified, with a starting amount of up to 75 ng for ChIP samples, using the DNA linear amplification method described previously [19].

#### Microarray hybridization

3 µg of aRNA produced from the linear amplification were used to label probe via the amino-allyl method as described on www.microarrays.org. Labeled probes were hybridized onto a yeast tiled oligonucleotide microarray [20] at 65°C for 16 h and washed as described on www.microarrays.org. The arrays were scanned at 5 micron resolution with an Axon Laboratories GenePix 4000B scanner running GenePix 5.1. Image analysis and data normalization were performed as previously described [19].

### Deep Sequencing Library Construction

ChIP DNA was treated with CIP (calf alkaline phosphatase NEB; in 1× NEB buffer 3, 0.25 U/µl CIP; 45 min at 37°C, reaction clean up with Qiagen MinElute spin columns). 20–150 ng of CIP treated ChIP DNA fragments were blunt ended and phosphorylated with the EPICENTRE End-it-Repair kit (1× buffer, 0.25 mM dNTPs,1 mM ATP, 1 µl/50 µl reaction of Enzyme mix) for 1 h at RT and cleaned up with Qiagen MinElute spin columns. Adenosine nucleotide overhangs were added using EPICENTRE exo-Klenow for 45 min at RT (with 0.2 mM dATP). Illumina genome sequencing adaptors were then ligated using the EPICENTRE Fast-Link ligation kit: 11.5 µl A tailed DNA eluted from a MinElute column was mixed with 1.5 µl 10× ligation buffer, 0.75 µl 10 mM ATP, 0.5 µl Illumina DNA adaptors, and 1 µl Ligase. The reaction was incubated for 1 h at RT and subsequently supplemented with 7.5 µl water, 1 µl 10× buffer, 0.5 µl 10 mM ATP, and 1 µl ligase, and incubated overnight at 16°C.

The ligation reaction was cleaned up with MinElute columns (with an additional wash step to eliminate all the excess adaptors) and the adaptor ligated fragments were amplified by PCR as follows: 0.5 µl of each Illumina genomic DNA sequencing primers, 10 µl 10× Pfx buffer 3 µl 10 mM dNTPs, 2 µl 50 mM MgSO4, and 1 µl Pfx DNA polymerase (Invitrogen) were added to 30 µl DNA template in a 100 µl reaction. The cycling parameters were: (1) 94°C, 2′; (2) 94°C, 15″; (3) 65°C, 1′; (4) 68°C, 30″; (5) repeat from (2) 17 times; (6) 68°C, 5′. The PCR product (200 to 300 bp in size) was gel purified from a 2% TAE agarose gel using the Freeze'N Squeeze columns (BioRad). Gel purified fragments were finally precipitated with Sodium acetate and Ethanol and pellets were resuspended (25 nM final concentration) in TE buffer and sent for SOLEXA sequencing at the UMass Worcester core deep sequencing facility.

### RNA Pol II ChIP and Microarray Hybridization

Cells were grown as described above. Cell pellets (∼10^9^ cells) were flash frozen after formaldehyde crosslinking (1%) and kept at −80°C overnight. Frozen cell pellets were resuspended in 300 µl cell braking buffer (100 mM Tris pH 7.9, 20% glycerol, 1× Sigma Protease inhibitors cocktail) and cell walls were broken down by bead beating using 400 µl of 0.5 mm zirconia/silica beads (BioSpec Products) in the BioSpec Mini-BeadBeater Model 8 three times for 1 min with 1 min pauses in between. Cell pellets (5 min max speed spin in refrigerated microcentrifuge) were then washed once and resuspended in 800 µl FA lysis buffer (with 1× Sigma Protease inhibitors cocktail). Chromatin was sheared by sonication in a cup sonicator (Branson, 50% pulse at strength 7 for 3.5 min) to 250–400 bp fragments.

The sheared chromatin suspension was pre-cleared with 100 µl Protein A-agarose slurry (IPA 400 HC RepliGen) at 4°C for 1 h. 100 µl of the pre-cleared solution was saved for the ChIP input sample and 7 µl of RNA Pol II antibody (abcam ab81859, lot #: 933570 and GR6094-1) was added to the rest and incubated overnight at 4°C with rotation. ChIP DNA isolation and amplification by TLAD was done as described previously [19].

2.5 μg of aRNA produced from the linear amplification were used to label probes via the amino-allyl method as described on www.microarrays.org. Labeled probes were hybridized onto a 4X44K yeast whole genome array (Agilent) at 65°C for 16 h. The arrays were scanned with the Agilent microarray scanner.

#### Data availability

Data are downloadable at http://www.umassmed.edu/bmp/faculty/rando.cfm and have been deposited in GEO (Accession # GSE28269).

#### Analysis

Raw sequencing data of HA and T7 libraries after the tag swap before release from arrest (0 generations), and at 1, 3, and 6 generations after release were uniquely mapped to the *S. cerevisiae* genome. Nucleosome positions were called from aggregated HA and T7 sequencing of the 3 generation sample using Template-Filtering [34]. For each nucleosome, we counted the number of supporting reads for each sample separately and calculated the ratio of HA reads to T7 reads at each nucleosome for each time point. Note, for the H4tailΔ data the T7 data quality was poor, so we used wild-type T7 sequences for this comparison.

For aggregated analyses such as those shown in Figure 2E or Figure 3, we calculated the median of the Log2 HA/T7 over the 1 kb starting at a given gene's transcription start site to provide a summary retention score per gene.

#### Model

Described in Text S1.

## Supporting Information

Figure S1 Recombination efficiency. Yeast were plated onto nonselective media and onto media selecting for the HA tag (linked to Hygro), before (t = 0) and after (t = o/n) inducing recombination. Roughly 2% of yeast fail to swap out the HA-Hygro insert.
(PDF)

Figure S2 HA/T7 at 3 and 6 generations after release. HA/T7 ratios (Log2) for individual nucleosomes are scatterplotted as indicated, showing good correlation but a slope <1 (red line), consistent with the background of nonswitching cells observed in Figure S1.
(PDF)

Figure S3 Validation of target genes. (A, C) Deep sequencing data (3 generations) for *SPA2* (A) and *BUD3* (C). (B, D) qPCR shown for the 5′ and 3′ ends of *SPA2* (B) and *BUD3* (D) at the indicated number of generations after tag-swap and release. Midlog refers to samples taken 3 h after the tag-swap was induced in exponentially growing cells that had not undergone a recent arrest. Note that only a fraction of all the cells had recombined out the old tag during the 3 h. qPCR amplicon locations are indicated under the gene annotation.
(PDF)

Figure S4 K means clustering of HA/T7 ratios. Log(2) HA/T7 ratios at 3 generations after release are shown as a heatmap for all genes, aligned by transcription start site (TSS) and clustered (K means, K = 5). Selected Gene Ontology (GO) enrichments for the various clusters are indicated to the right of the clusters.
(TIF)

Figure S5 Histone retention anticorrelates with turnover. (A) Average profiles for the 5 clusters from Figure S4 are plotted relative to TSS-aligned coding regions. (B) Replication-independent turnover (Z score, [15]) was averaged for 5′ CDS, mid-CDS, and 3′ CDS for all genes in each cluster. Note that Cluster 2, which exhibits a somewhat 3′-shifted peak of HA/T7 relative to Clusters 3–5 (see A), consists of genes with relatively high 5′ turnover, which presumably explains the downstream location of the HA/T7 peak in this cluster.
(PDF)

Figure S6 Histone retention anticorrelates with transcription frequency. (A–B) As in Figure 2B–C. (C) As in (B), but using “transcription frequency” defined in Holstege et al. [71] rather than Pol2 ChIP. (D) As in Figure 2F, but using Holstege et al. data rather than Pol2 ChIP data.
(TIF)

Figure S7 H3 retention at subtelomeric genes. Median HA/T7 over the 5′ 1 kb of all genes is plotted versus distance from the closest telomere, with an 80 gene running window average shown in red. No specific enrichment of H3-HA is observed near telomeres. Similar results are found for repetitive subtelomeric genes (unpublished data).
(PDF)

Figure S8 Mutant analysis of ancestral H3 retention. (A) 5′/3′ ratio at *SPA2* or *BUD3* were measured by q-PCR for the various mutants 3 generations after release or after one round of replication arrested in G2/M. 5′/3′ ratio relative to wild-type level is plotted on the *y*-axis. Mutants are as indicated, with *swd1*, *swd3* referring to an average of single replicates with each individual mutant and *scc1* referring to one experiment using a *pGAL1-SCC1* allele that was shut off by release into glucose media after the tag switch (leading to a G2/M arrest). Average of mutant/wt, ± S.E.M. (*n* = 2). Swd1 and Swd3 are components of the Set1 complex, which methylates H3K4. Set2 is the H3K36 methylase and Scc1 is part of the cohesin complex. (B) 5′/3′ ratio at *SPA2* or *BUD3* were measured by q-PCR for the various mutants after one round of replication arrested in G2/M. 5′/3′ ratio is plotted on the *y*-axis with wild-type set to 1. Average ± S.E.M. (*n* = 2). H4K5,12R is a mutant in which two of the acetylatable lysines of the H4 tail have been replaced by arginine, mimicking the unacetylated state. Rtt109 is a histone acetyltransferase that binds to Asf1 and acetylates new histone H3 on K56 [72]. Nhp6a/b are non-essential HMGB proteins [73] that are required for FACT activity. The FACT core subunits Spt16 and Pob3 are essential, precluding us from testing their functions in ancestral histone inheritance. (C) As in (A–B), for the indicated PCNA (*POL30*) point mutants. Average of mutant/wt, ± S.E.M. (*n* = 2).
(PDF)

Figure S9 HA retention on length-normalized genes. (A) All genes were normalized to a length of 1, and genes are ordered by Pol2 ChIP. Log2 HA/T7 ratios are shown as a heatmap. (B) Running window average of data from (A). Note the 5′ shift of the downstream edge of the HA/T7 peak with increasing transcription rates. (C) Pol2 ChIP for genes as ordered in (A–B). (D) Averages for all length-normalized genes grouped into 6 bins of transcription level.
(PDF)

Figure S10 5′ accumulation of ancestral histones occurs in the absence of nutrient stress. (A) Yeast carrying the HA/T7 recombination cassette were grown continuously in YPD, then were treated with β-estradiol for 6 h to induce recombination. HA and T7 ChIPs were carried out after 6 h and deep sequenced, and normalized HA/T7 ratios were calculated. Here, genes are ordered in 5 clusters as in Figure S4. (B) Averaged data for cells arrested, switched, and released for 3 generations (“3 gen”) are shown alongside data from the midlog switch. Note that 5′ accumulation occurs in both conditions but to a lesser extent in the midlog swap. This is an expected result of the heterogeneity of switch timing in midlog cells—only 65% of yeast have completed recombination after 3 h, with 85% complete by 6 h (unpublished data), meaning that the midlog switch represents a mixture of cells that have recently swapped tags with those that swapped tags ∼1–3 generations prior. (C) As in (B), for intermediate and long genes.
(TIF)

Figure S11 A quantitative model accurately captures ancestral H3 retention patterns. Model predictions (red lines) and data (blue lines) for HA/T7 ratios at 1, 3, and 6 generations after tag swap are shown for 1–2 kb genes (A) and >2 kb genes (B). The model performs better on longer genes than on short genes.
(PDF)

Figure S12 Passback correlates with transcription rate. Estimated passback parameters for each gene were compared to Pol2 ChIP values for each gene. Scatterplot is colored by density of points—red indicates greater density of points. White line indicates linear fit to dataset, R = 0.12.
(PDF)

Figure S13 Short genes and 3′ ends are poorly predicted by the model. Genes are ordered by length, and the difference between model predictions for 3 generations and actual data are shown as a heatmap—yellow indicates the model predicts excessive old nucleosome loss, or lower HA/T7 ratios than measured. Notably, the +N nucleosome is universally predicted to lose more H3-HA than is measured. This is almost certainly a consequence of the fact that our model considers all genes in isolation—there is no way for histones to spread onto the 3′ end of a gene from adjacent genomic loci in this model, although this likely occurs in vivo.
(PDF)

Figure S14 The H4 N-terminal tail qualitatively changes the relationship between transcription and ancestral H3 retention. (A) Tag swap strains carrying an H4 N-terminal tail deletion were processed as in Figure 2B. Genes are ordered by the median HA/T7 over the 5′ 1 kb. (B) Pol2 ChIP was carried out in the H4 tail deletion strain 2 generations after release from arrest. Data here show an 80 gene running window average of Pol2 ChIP level per gene. (C) As in Figure 2D. Genes with high HA/T7 ratios in the H4 tail deletion mutant actually tend to be slightly *more* enriched for Pol2 than those with low HA/T7 levels, the opposite of what is seen in wild-type (although it is important to note that the correlation with Pol2 levels in this mutant is very weak—note that the scale bar for Pol2 ChIP here ranges from −0.1 to 0.1, whereas the scale bar in Figure 2D ranges from −0.2 to 1). Thus, we can conclude with confidence that the effects of the H4 tail deletion do not simply result from extensive transcription reprogramming in these mutants, since the relationship between Pol2 and H3-HA retention *qualitatively* changes in this mutant.
(TIF)

Figure S15 Replication time has subtle effects of ancestral H3 patterns. (A) Data for the 20% earliest and 20% latest-replicating [74] genes is averaged as indicated. (B) As in (A), with gene lengths normalized to one.
(PDF)

Figure S16 CAF-1 mutation affects far-5′ end levels of ancestral H3. Averaged data for clusters 4 and 5 (Figure S4) are shown for wild-type and *cac1Δ*.
(PDF)

Figure S17 Ancestral H3 retention and histone modification patterns. Modification levels [52] compared to ancestral H3 retention. For each modification, genes were grouped into high, middle, and low HA/T7 (based on the 5′-most 1 kb median HA/T7), and for each group of genes modifications were averaged for 5-CDS (“5”), mid-CDS (“m”), and 3′-CDS (“3”) as previously described [15],[19]. Groupings are indicated for H3K9ac and for H3K79me3 and are the same for the other three modifications.
(PDF)

Figure S18 Role for histone movement in shaping modification landscapes. Schematic for retrograde histone movement in shaping histone modification landscapes. Initial 5′ (green) and 3′ (purple) histone modification states could, in the absence of erasing enzymes, eventually give rise to skewed distributions via retrograde motion of old histones bearing 3′ modifications such as H3K36me3 (purple). Importantly, after a few cell divisions old histones on average constitute only a minor fraction of all histones at any given locus (e.g., see Figure S3B, D). Modifications on ancestral histones will therefore make subtle contributions to overall average modification patterns.
(PDF)

Table S1 HA/T7 ratios for 3 generations. Log2(HA/T7) for all genes is shown at varying distances with respect to the TSS. Genes are sorted according to the median HA/T7 over the 5′ 1 kb.
(XLSX)

Table S2 Enrichments of genesets for high or low retrograde passback values. A variety of gene sets were searched for enrichment of relatively high or low values for model estimates of lateral nucleosome movement. Negative KS values indicate large 3′ to 5′ lateral movements; positive values indicate the converse.
(XLSX)

Table S3 Strain list. Genotypes of strains used in this study.
(XLS)

Table S4 Primers used. Primer sequences.
(XLS)

Text S1 Quantitative model for multigenerational histone dynamics. Detailed description of model shown in Figures 5 and 6.
(PDF)
