# Supplementary figures and images for: Patterns and Mechanisms of Ancestral Histone Protein Inheritance in Budding Yeast
Source: PLoS Biol. 2011 Jun 7;9(6):e1001075. doi: 10.1371/journal.pbio.1001075 (PMC3110181; doi:10.1371/journal.pbio.1001075)

Figure S1

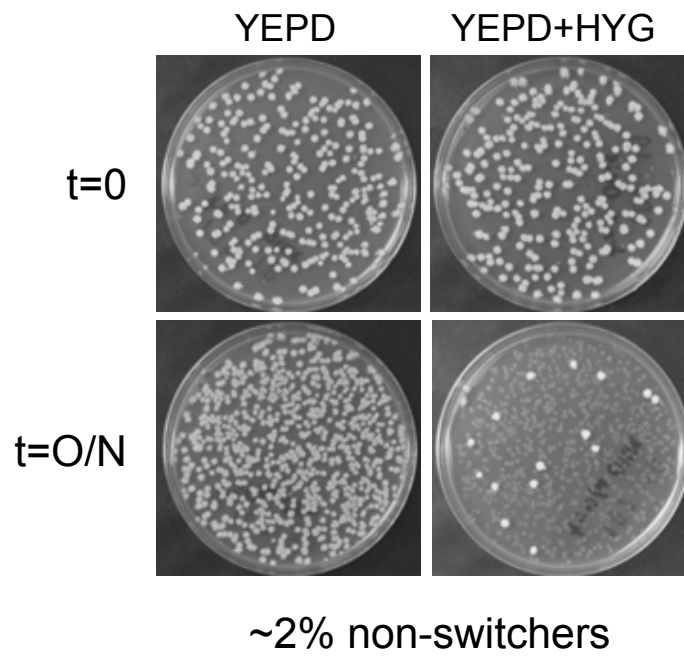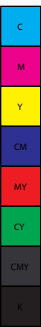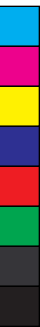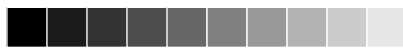

Supplement: Figure S1 — Recombination efficiency. Yeast were plated onto nonselective media and onto media selecting for the HA tag (linked to Hygro), before (t = 0) and after (t = o/n) inducing recombination. Roughly 2% of yeast fail to swap out the HA-Hygro insert. (PDF) [file pbio.1001075.s001.pdf]

Figure S2

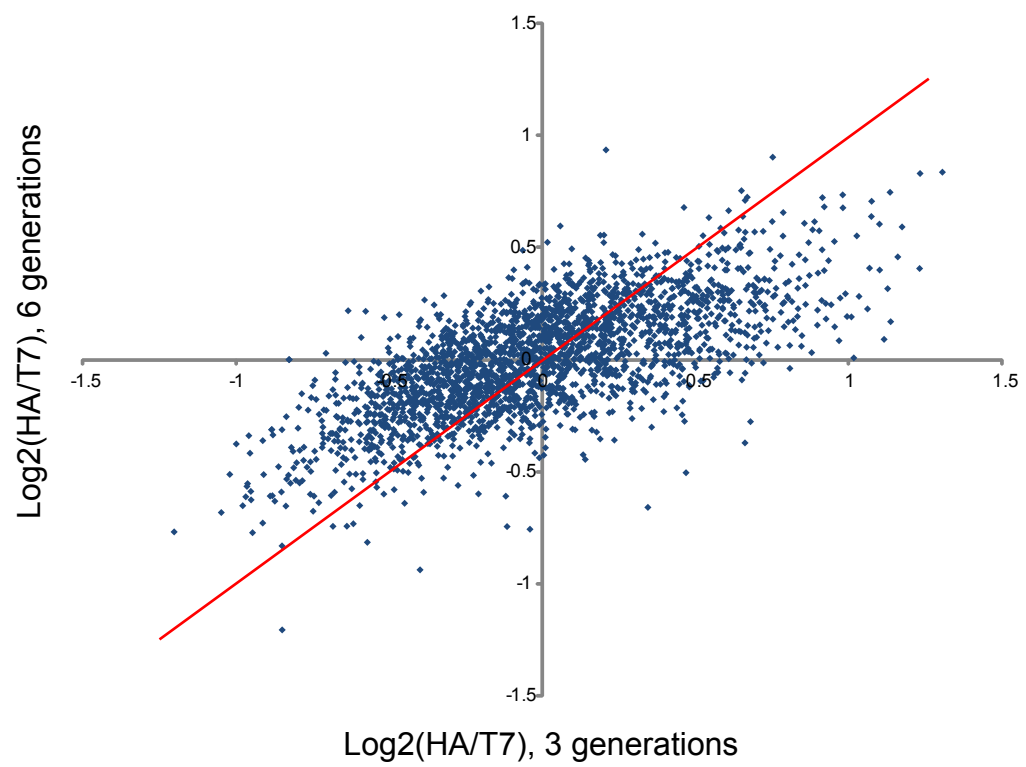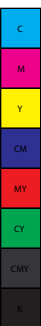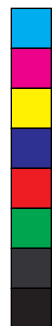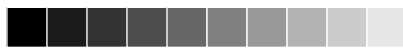

Supplement: Figure S2 — HA/T7 at 3 and 6 generations after release. HA/T7 ratios (Log2) for individual nucleosomes are scatterplotted as indicated, showing good correlation but a slope <1 (red line), consistent with the background of nonswitching cells observed in Figure S1. (PDF) [file pbio.1001075.s002.pdf]

Figure S3

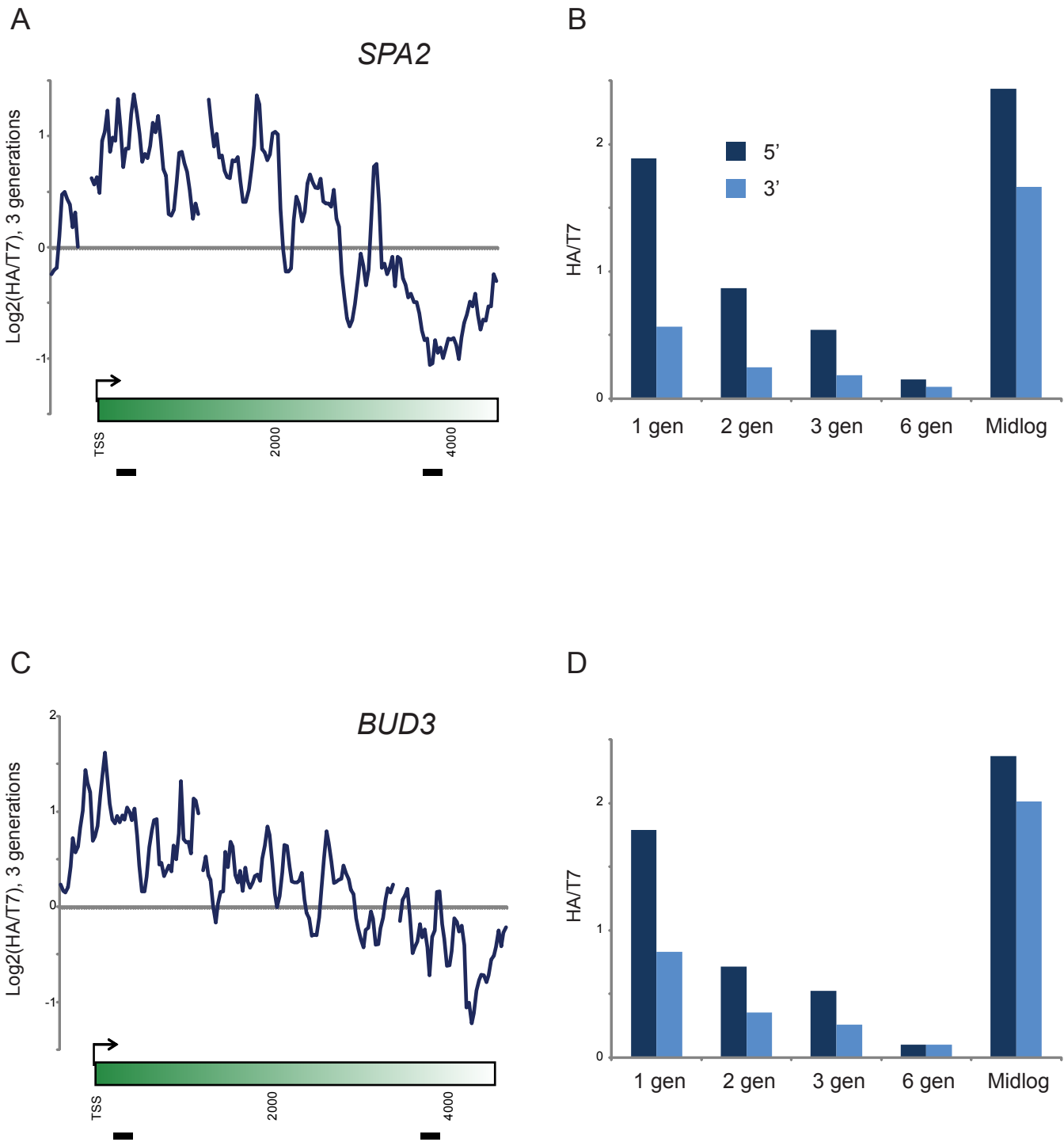

Supplement: Figure S3 — Validation of target genes. (A, C) Deep sequencing data (3 generations) for SPA2 (A) and BUD3 (C). (B, D) qPCR shown for the 5′ and 3′ ends of SPA2 (B) and BUD3 (D) at the indicated number of generations after tag-swap and release. Midlog refers to samples taken 3 h after the tag-swap was induced in exponentially growing cells that had not undergone a recent arrest. Note that only a fraction of all the cells had recombined out the old tag during the 3 h. qPCR amplicon locations are indicated under the gene annotation. (PDF) [file pbio.1001075.s003.pdf]

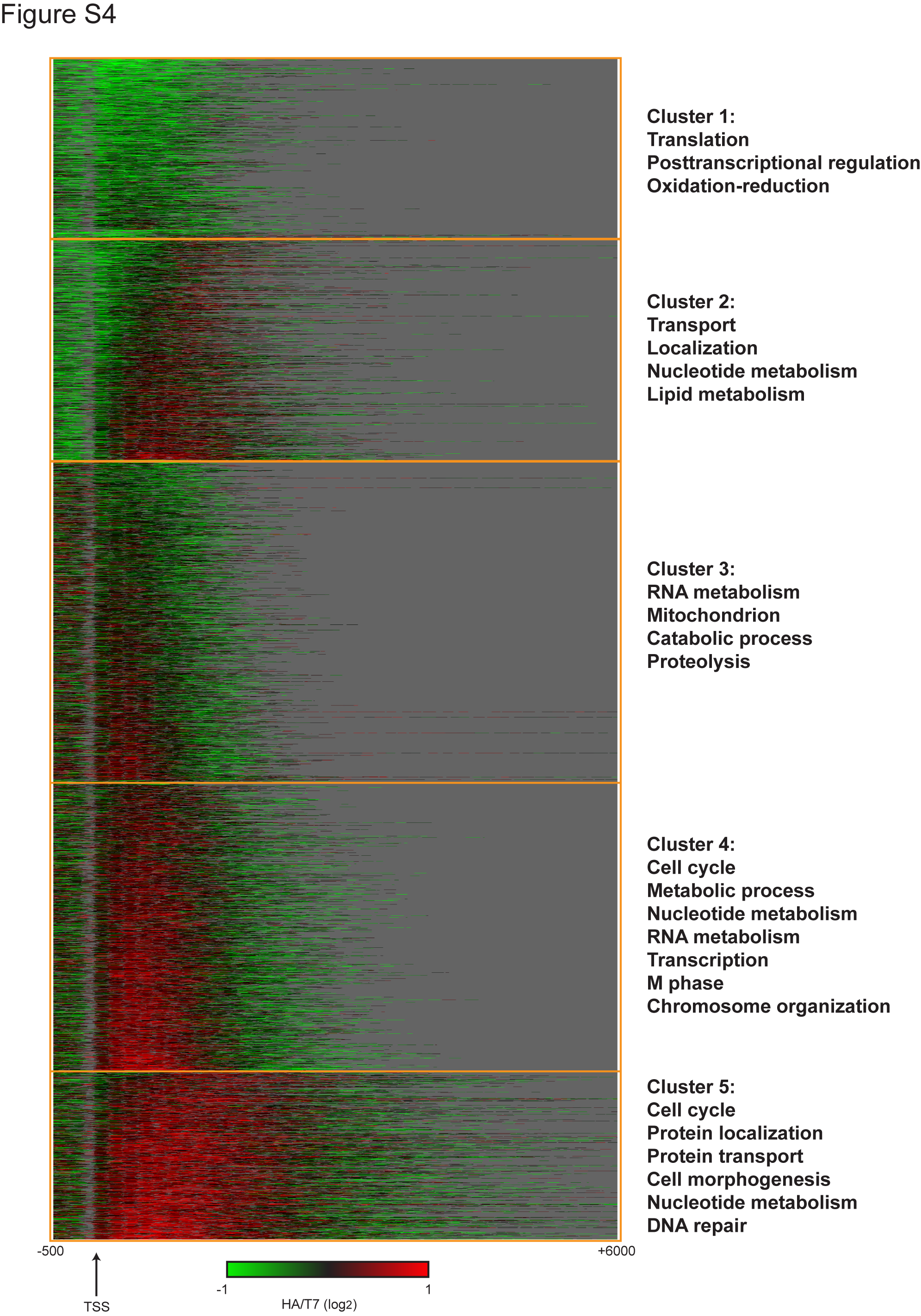

Supplement: Figure S4 — K means clustering of HA/T7 ratios. Log(2) HA/T7 ratios at 3 generations after release are shown as a heatmap for all genes, aligned by transcription start site (TSS) and clustered (K means, K = 5). Selected Gene Ontology (GO) enrichments for the various clusters are indicated to the right of the clusters. (TIF) [file pbio.1001075.s004.tif]

Figure S5

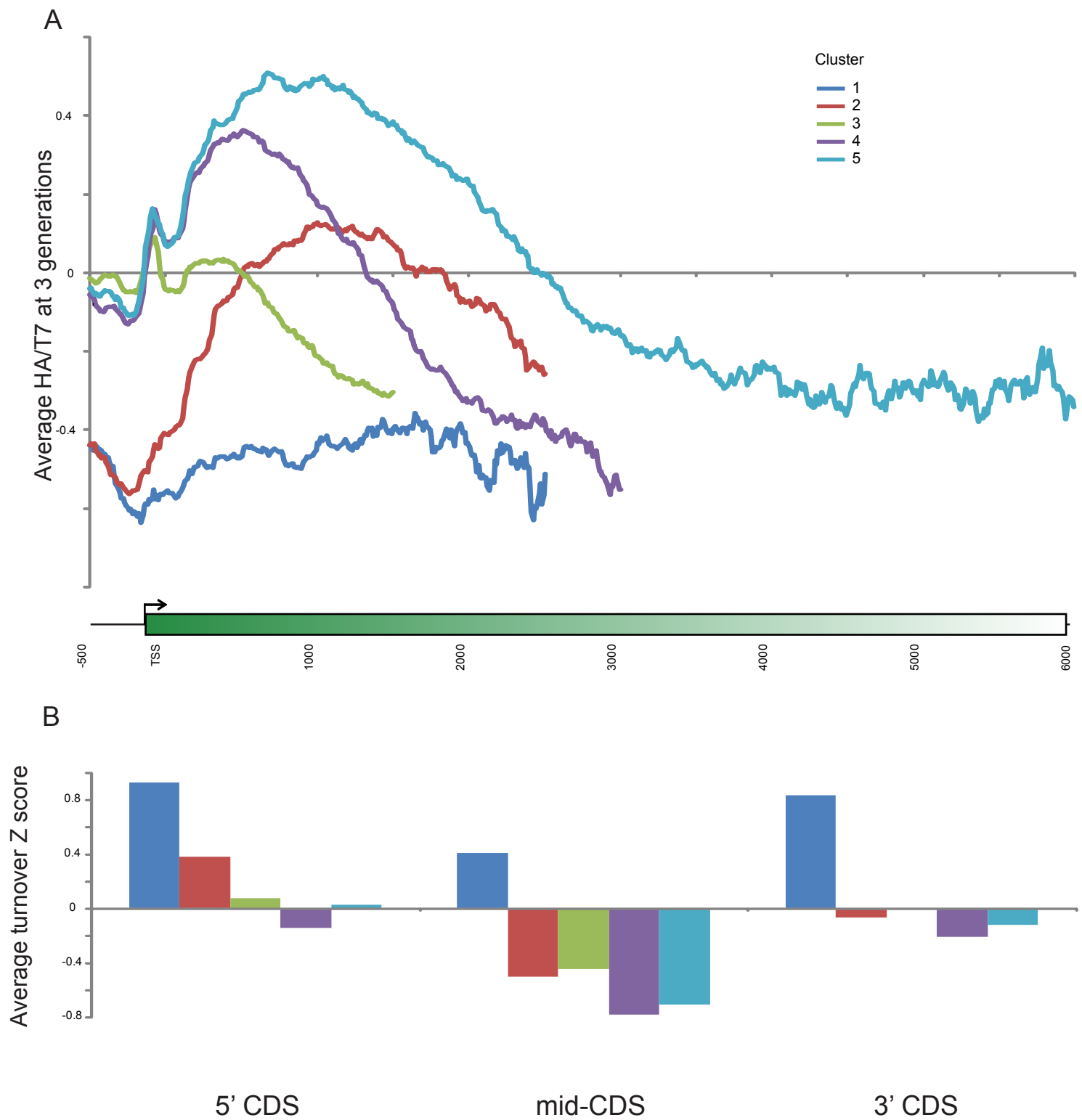

Supplement: Figure S5 — Histone retention anticorrelates with turnover. (A) Average profiles for the 5 clusters from Figure S4 are plotted relative to TSS-aligned coding regions. (B) Replication-independent turnover (Z score, [15]) was averaged for 5′ CDS, mid-CDS, and 3′ CDS for all genes in each cluster. Note that Cluster 2, which exhibits a somewhat 3′-shifted peak of HA/T7 relative to Clusters 3–5 (see A), consists of genes with relatively high 5′ turnover, which presumably explains the downstream location of the HA/T7 peak in this cluster. (PDF) [file pbio.1001075.s005.pdf]

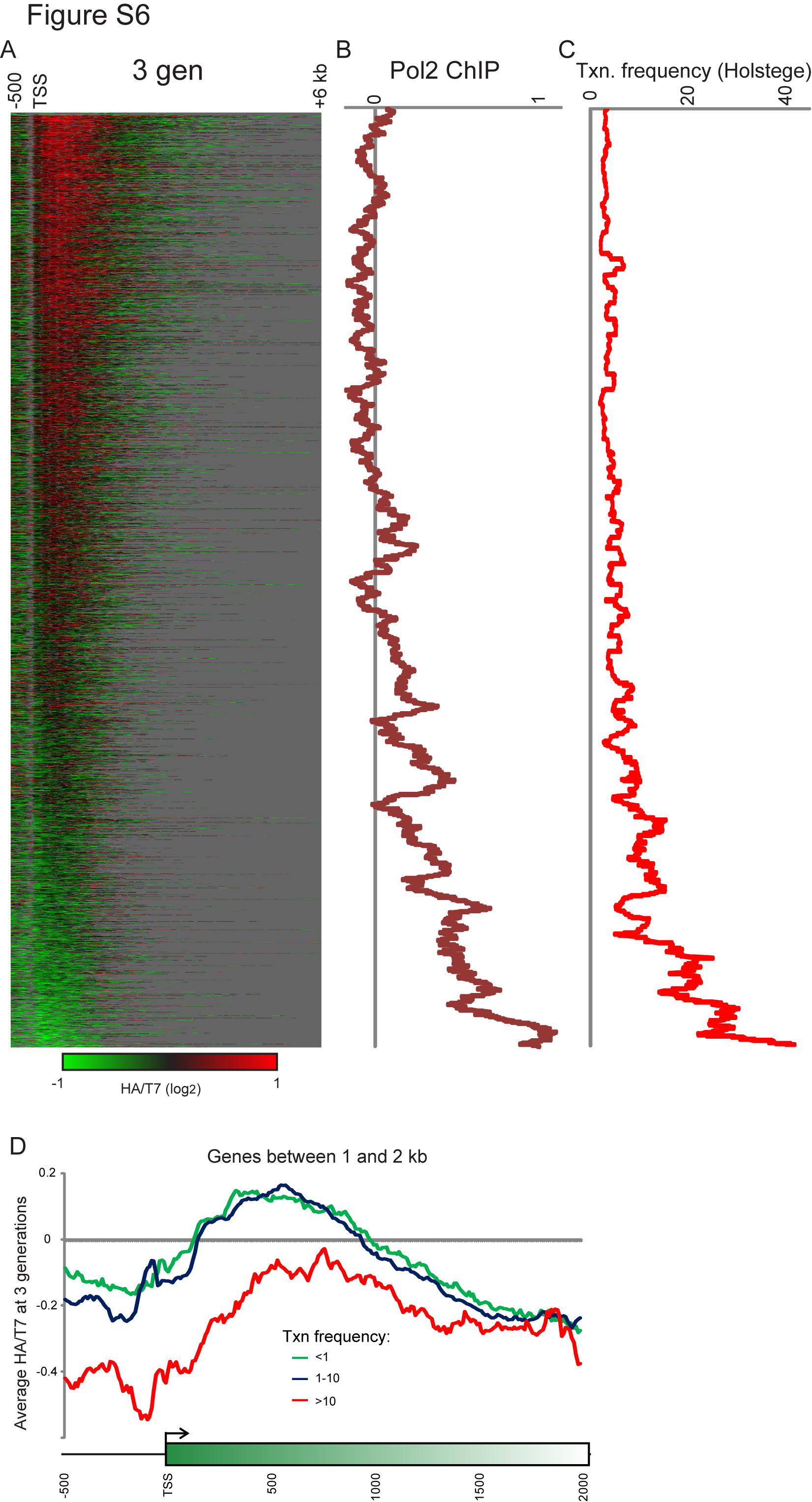

Supplement: Figure S6 — Histone retention anticorrelates with transcription frequency. (A–B) As in Figure 2B–C. (C) As in (B), but using “transcription frequency” defined in Holstege et al. [71] rather than Pol2 ChIP. (D) As in Figure 2F, but using Holstege et al. data rather than Pol2 ChIP data. (TIF) [file pbio.1001075.s006.tif]

Figure S7

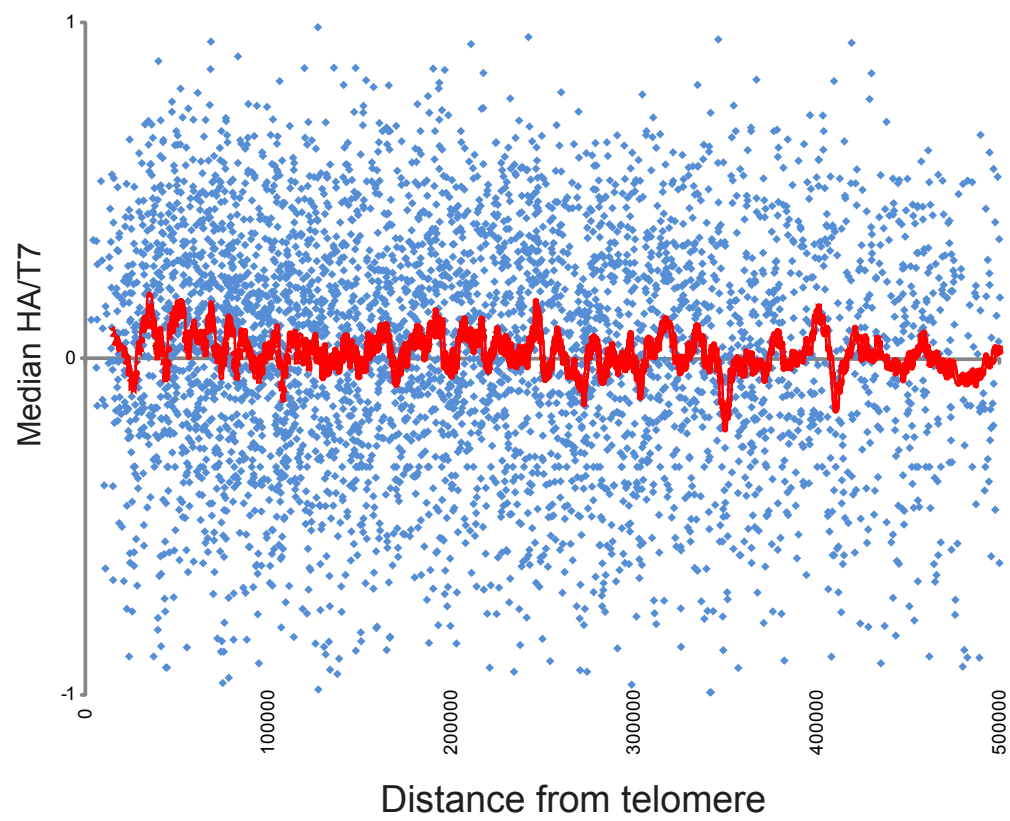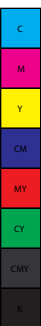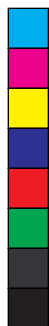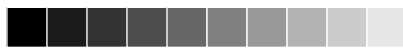

Supplement: Figure S7 — H3 retention at subtelomeric genes. Median HA/T7 over the 5′ 1 kb of all genes is plotted versus distance from the closest telomere, with an 80 gene running window average shown in red. No specific enrichment of H3-HA is observed near telomeres. Similar results are found for repetitive subtelomeric genes (unpublished data). (PDF) [file pbio.1001075.s007.pdf]

Figure S8

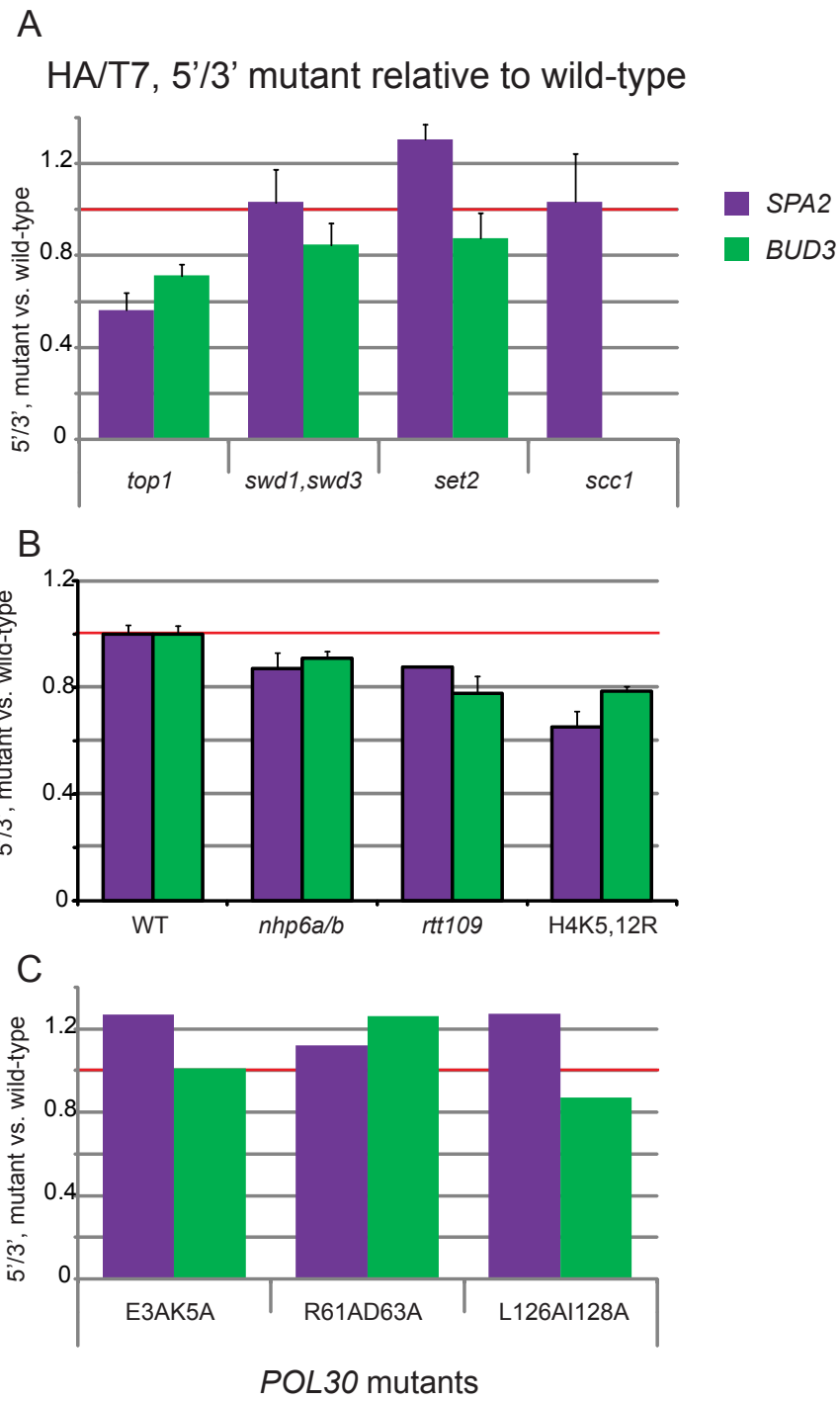

Supplement: Figure S8 — Mutant analysis of ancestral H3 retention. (A) 5′/3′ ratio at SPA2 or BUD3 were measured by q-PCR for the various mutants 3 generations after release or after one round of replication arrested in G2/M. 5′/3′ ratio relative to wild-type level is plotted on the y-axis. Mutants are as indicated, with swd1, swd3 referring to an average of single replicates with each individual mutant and scc1 referring to one experiment using a pGAL1-SCC1 allele that was shut off by release into glucose media after the tag switch (leading to a G2/M arrest). Average of mutant/wt, ± S.E.M. (n = 2). Swd1 and Swd3 are components of the Set1 complex, which methylates H3K4. Set2 is the H3K36 methylase and Scc1 is part of the cohesin complex. (B) 5′/3′ ratio at SPA2 or BUD3 were measured by q-PCR for the various mutants after one round of replication arrested in G2/M. 5′/3′ ratio is plotted on the y-axis with wild-type set to 1. Average ± S.E.M. (n = 2). H4K5,12R is a mutant in which two of the acetylatable lysines of the H4 tail have been replaced by arginine, mimicking the unacetylated state. Rtt109 is a histone acetyltransferase that binds to Asf1 and acetylates new histone H3 on K56 [72]. Nhp6a/b are non-essential HMGB proteins [73] that are required for FACT activity. The FACT core subunits Spt16 and Pob3 are essential, precluding us from testing their functions in ancestral histone inheritance. (C) As in (A–B), for the indicated PCNA (POL30) point mutants. Average of mutant/wt, ± S.E.M. (n = 2). (PDF) [file pbio.1001075.s008.pdf]

Figure S9

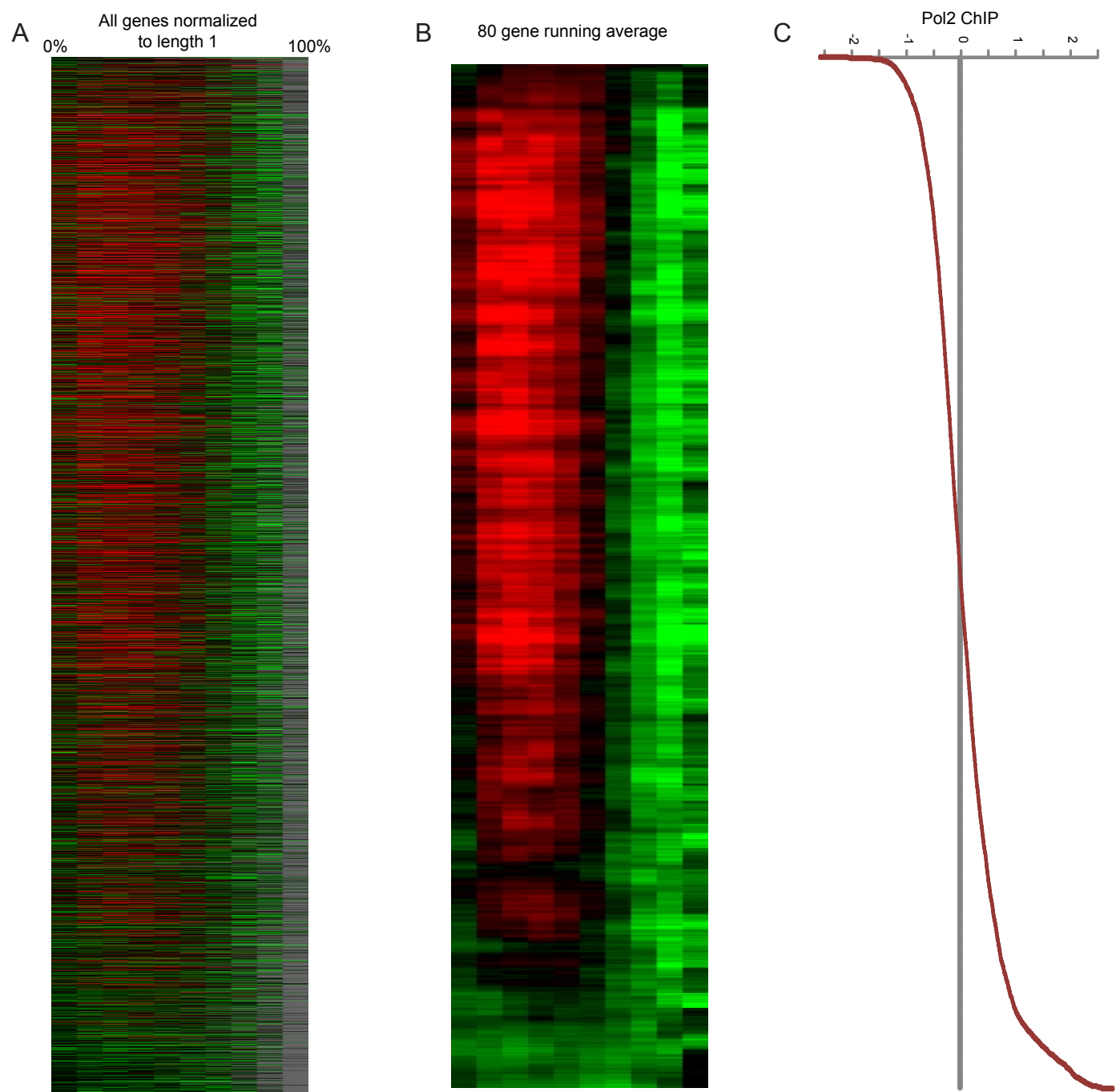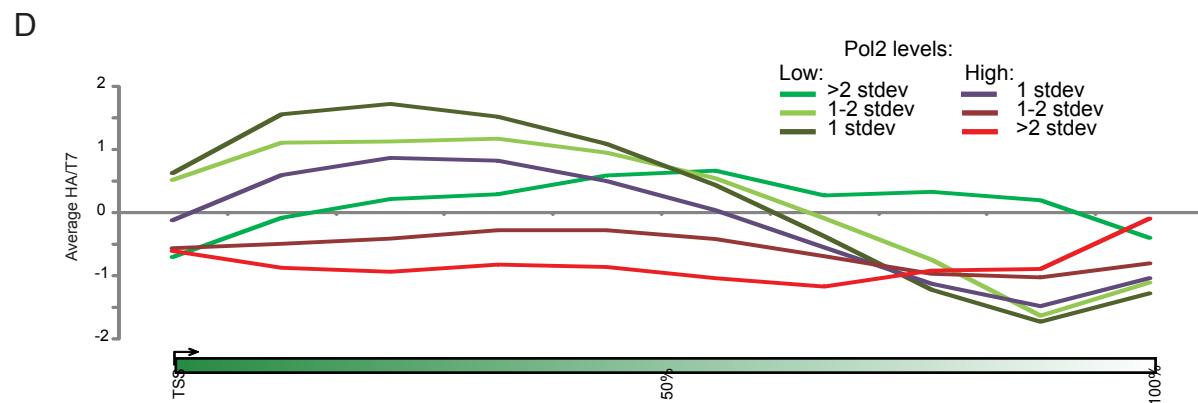

Supplement: Figure S9 — HA retention on length-normalized genes. (A) All genes were normalized to a length of 1, and genes are ordered by Pol2 ChIP. Log2 HA/T7 ratios are shown as a heatmap. (B) Running window average of data from (A). Note the 5′ shift of the downstream edge of the HA/T7 peak with increasing transcription rates. (C) Pol2 ChIP for genes as ordered in (A–B). (D) Averages for all length-normalized genes grouped into 6 bins of transcription level. (PDF) [file pbio.1001075.s009.pdf]

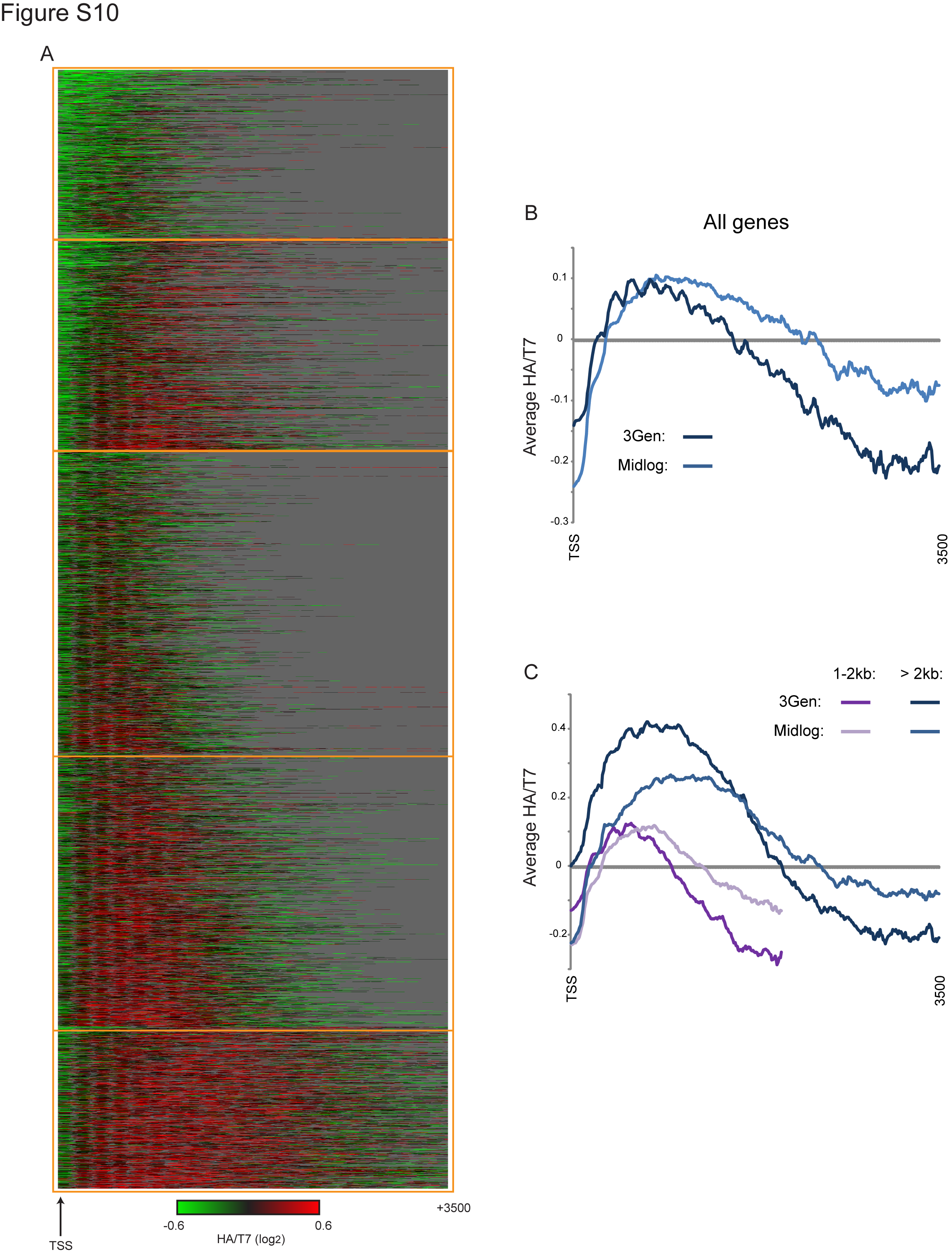

Supplement: Figure S10 — 5′ accumulation of ancestral histones occurs in the absence of nutrient stress. (A) Yeast carrying the HA/T7 recombination cassette were grown continuously in YPD, then were treated with β-estradiol for 6 h to induce recombination. HA and T7 ChIPs were carried out after 6 h and deep sequenced, and normalized HA/T7 ratios were calculated. Here, genes are ordered in 5 clusters as in Figure S4. (B) Averaged data for cells arrested, switched, and released for 3 generations (“3 gen”) are shown alongside data from the midlog switch. Note that 5′ accumulation occurs in both conditions but to a lesser extent in the midlog swap. This is an expected result of the heterogeneity of switch timing in midlog cells—only 65% of yeast have completed recombination after 3 h, with 85% complete by 6 h (unpublished data), meaning that the midlog switch represents a mixture of cells that have recently swapped tags with those that swapped tags ∼1–3 generations prior. (C) As in (B), for intermediate and long genes. (TIF) [file pbio.1001075.s010.tif]

Figure S11

A

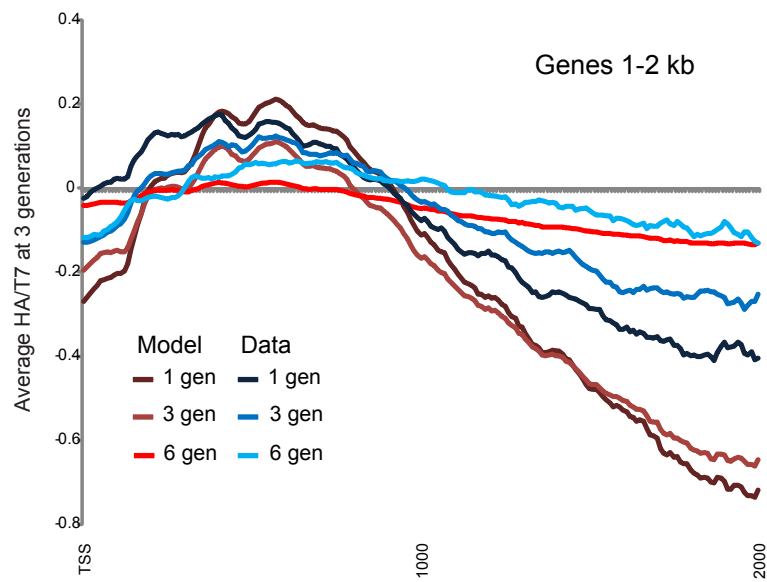

B

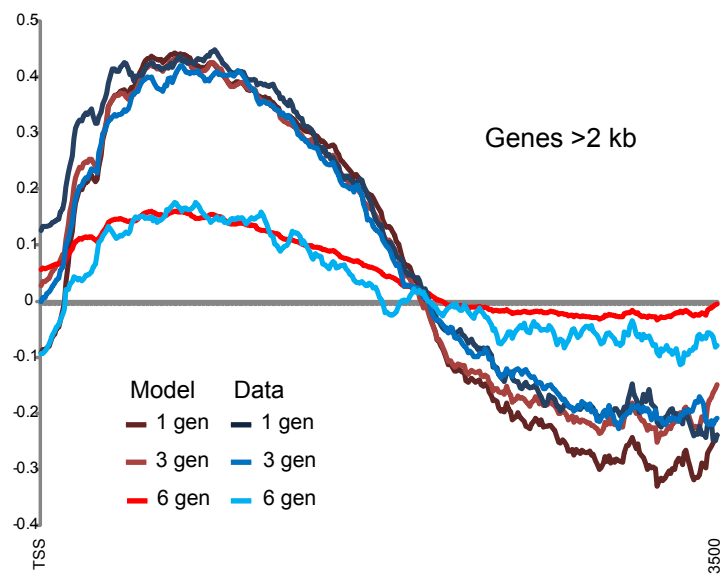

Supplement: Figure S11 — A quantitative model accurately captures ancestral H3 retention patterns. Model predictions (red lines) and data (blue lines) for HA/T7 ratios at 1, 3, and 6 generations after tag swap are shown for 1–2 kb genes (A) and >2 kb genes (B). The model performs better on longer genes than on short genes. (PDF) [file pbio.1001075.s011.pdf]

Figure S12

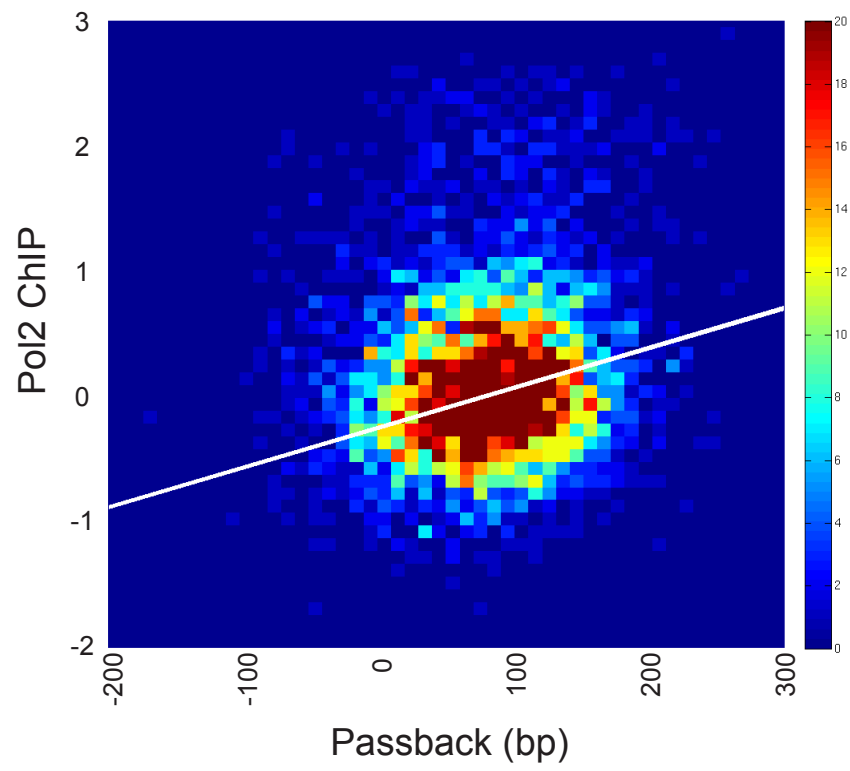

Supplement: Figure S12 — Passback correlates with transcription rate. Estimated passback parameters for each gene were compared to Pol2 ChIP values for each gene. Scatterplot is colored by density of points—red indicates greater density of points. White line indicates linear fit to dataset, R = 0.12. (PDF) [file pbio.1001075.s012.pdf]

Figure S13

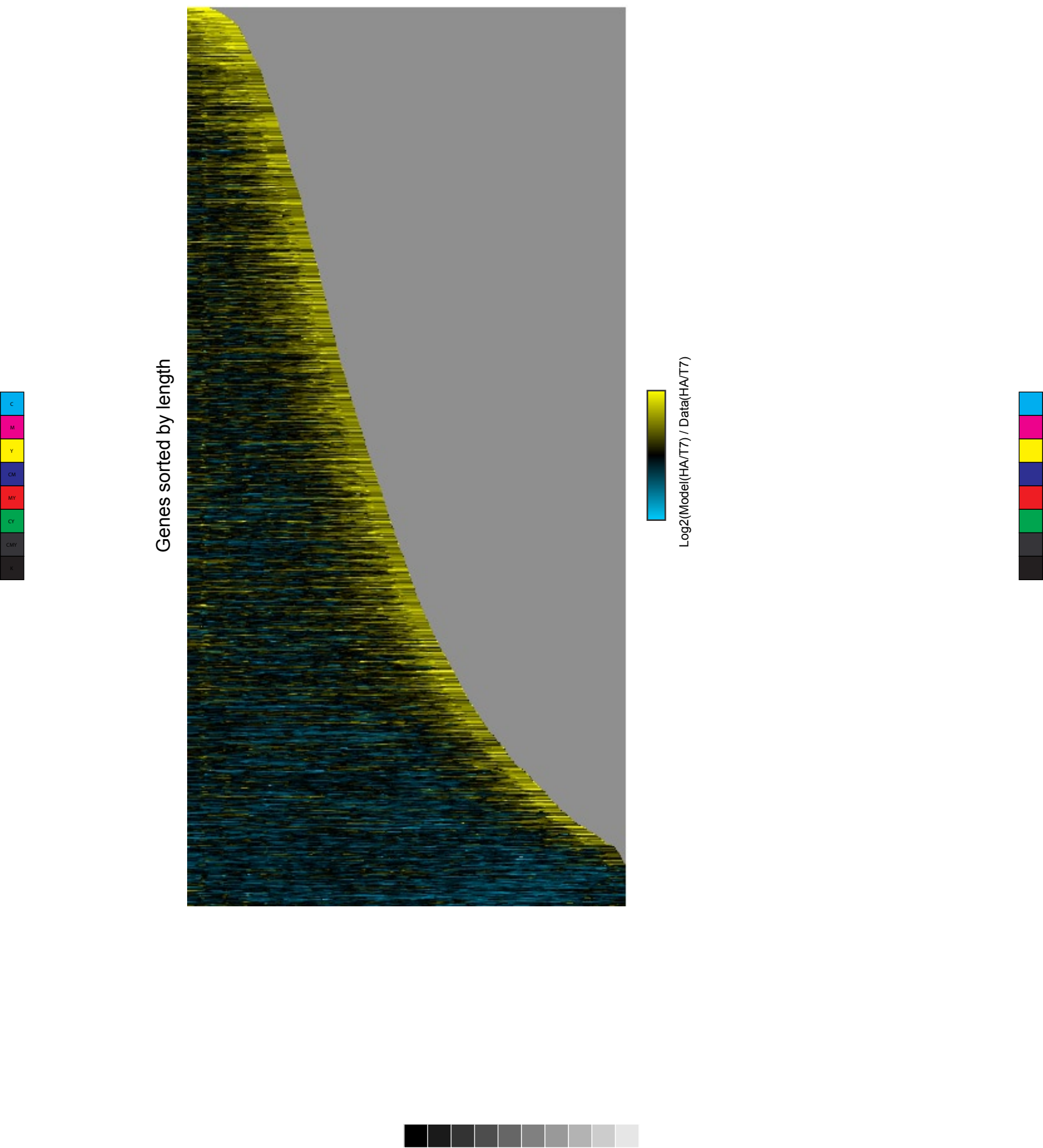

Supplement: Figure S13 — Short genes and 3′ ends are poorly predicted by the model. Genes are ordered by length, and the difference between model predictions for 3 generations and actual data are shown as a heatmap—yellow indicates the model predicts excessive old nucleosome loss, or lower HA/T7 ratios than measured. Notably, the +N nucleosome is universally predicted to lose more H3-HA than is measured. This is almost certainly a consequence of the fact that our model considers all genes in isolation—there is no way for histones to spread onto the 3′ end of a gene from adjacent genomic loci in this model, although this likely occurs in vivo. (PDF) [file pbio.1001075.s013.pdf]

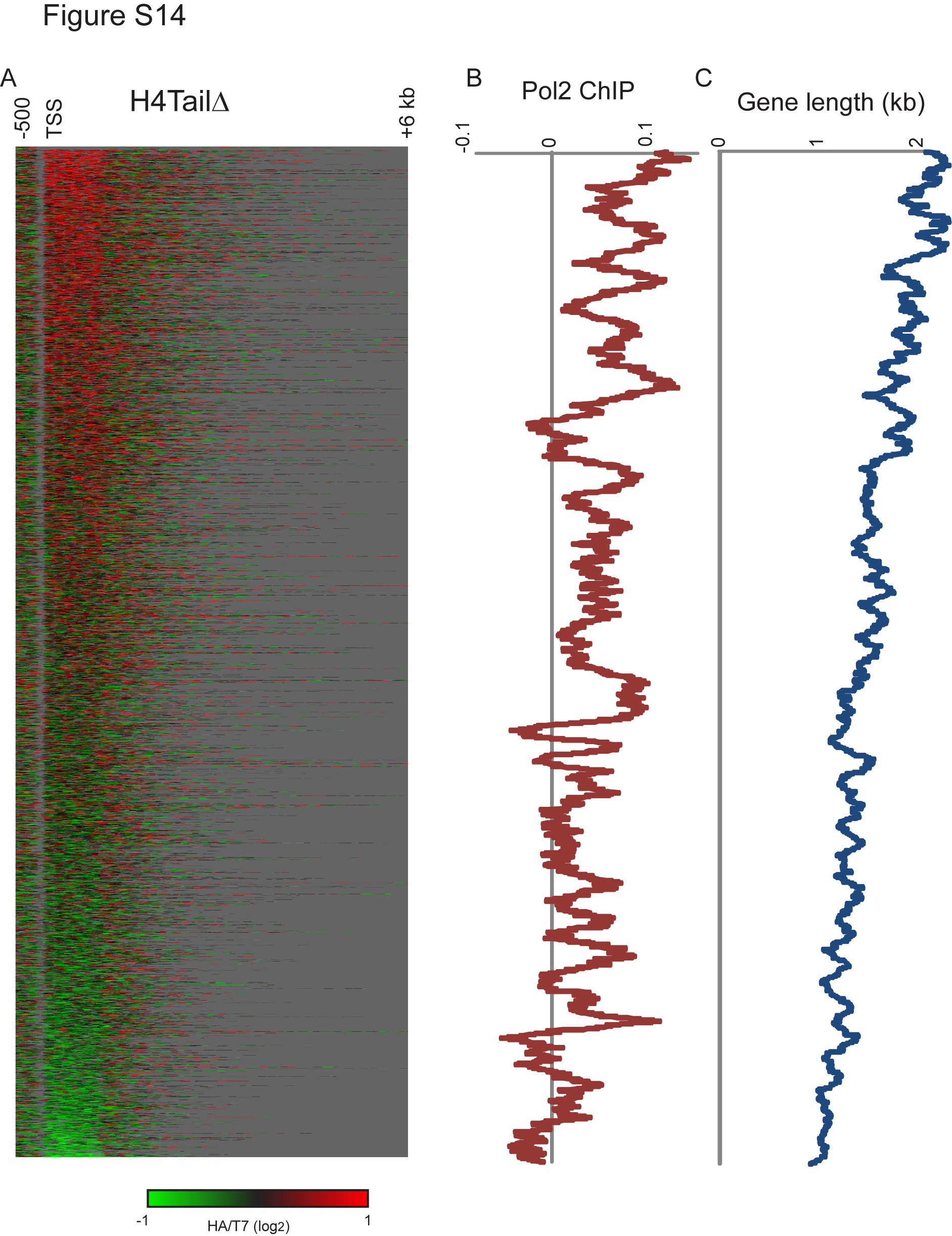

Supplement: Figure S14 — The H4 N-terminal tail qualitatively changes the relationship between transcription and ancestral H3 retention. (A) Tag swap strains carrying an H4 N-terminal tail deletion were processed as in Figure 2B. Genes are ordered by the median HA/T7 over the 5′ 1 kb. (B) Pol2 ChIP was carried out in the H4 tail deletion strain 2 generations after release from arrest. Data here show an 80 gene running window average of Pol2 ChIP level per gene. (C) As in Figure 2D. Genes with high HA/T7 ratios in the H4 tail deletion mutant actually tend to be slightly more enriched for Pol2 than those with low HA/T7 levels, the opposite of what is seen in wild-type (although it is important to note that the correlation with Pol2 levels in this mutant is very weak—note that the scale bar for Pol2 ChIP here ranges from −0.1 to 0.1, whereas the scale bar in Figure 2D ranges from −0.2 to 1). Thus, we can conclude with confidence that the effects of the H4 tail deletion do not simply result from extensive transcription reprogramming in these mutants, since the relationship between Pol2 and H3-HA retention qualitatively changes in this mutant. (TIF) [file pbio.1001075.s014.tif]

Figure S15

A

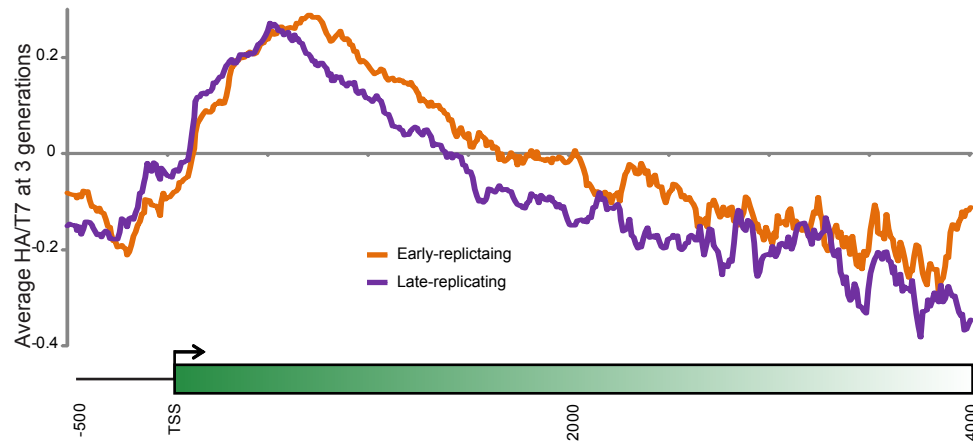

B

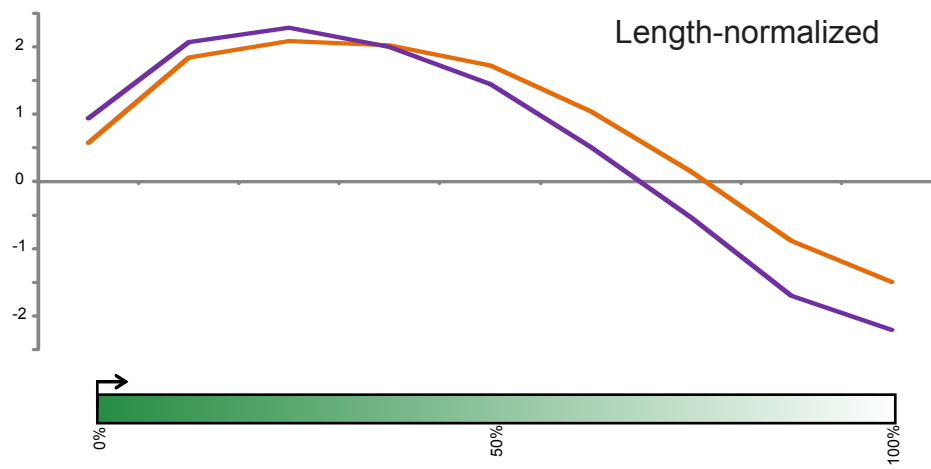

Supplement: Figure S15 — Replication time has subtle effects of ancestral H3 patterns. (A) Data for the 20% earliest and 20% latest-replicating [74] genes is averaged as indicated. (B) As in (A), with gene lengths normalized to one. (PDF) [file pbio.1001075.s015.pdf]

Figure S16

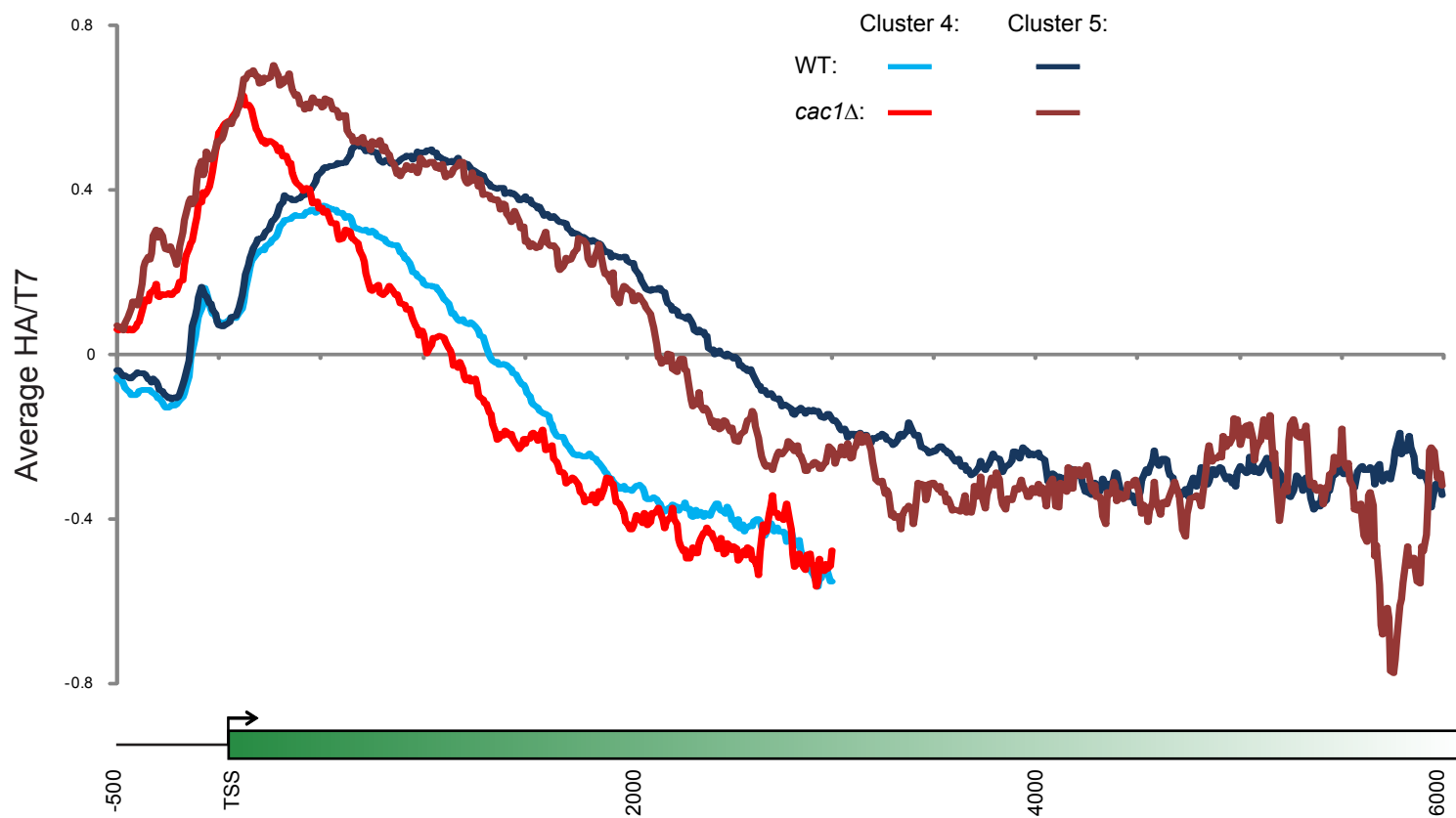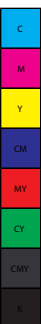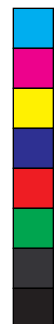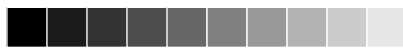

Supplement: Figure S16 — CAF-1 mutation affects far-5′ end levels of ancestral H3. Averaged data for clusters 4 and 5 (Figure S4) are shown for wild-type and cac1Δ. (PDF) [file pbio.1001075.s016.pdf]

Figure S17

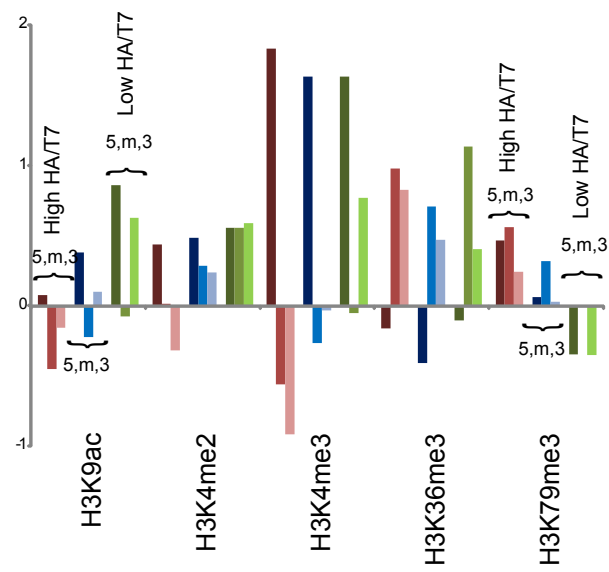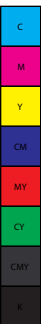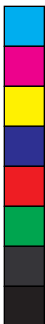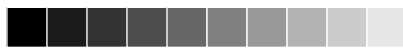

Supplement: Figure S17 — Ancestral H3 retention and histone modification patterns. Modification levels [52] compared to ancestral H3 retention. For each modification, genes were grouped into high, middle, and low HA/T7 (based on the 5′-most 1 kb median HA/T7), and for each group of genes modifications were averaged for 5-CDS (“5”), mid-CDS (“m”), and 3′-CDS (“3”) as previously described [15],[19]. Groupings are indicated for H3K9ac and for H3K79me3 and are the same for the other three modifications. (PDF) [file pbio.1001075.s017.pdf]

Figure S18

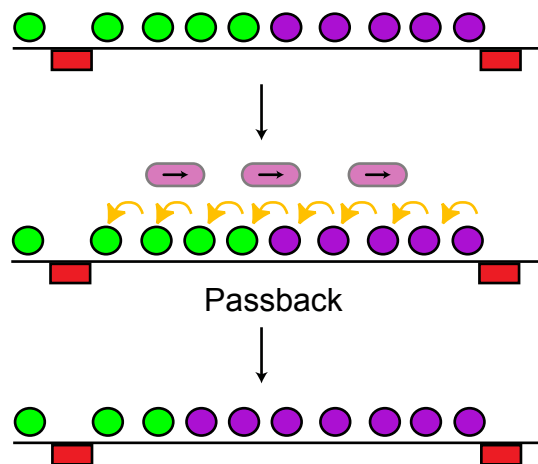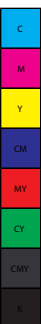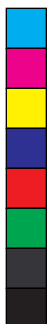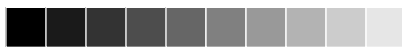

Supplement: Figure S18 — Role for histone movement in shaping modification landscapes. Schematic for retrograde histone movement in shaping histone modification landscapes. Initial 5′ (green) and 3′ (purple) histone modification states could, in the absence of erasing enzymes, eventually give rise to skewed distributions via retrograde motion of old histones bearing 3′ modifications such as H3K36me3 (purple). Importantly, after a few cell divisions old histones on average constitute only a minor fraction of all histones at any given locus (e.g., see Figure S3B, D). Modifications on ancestral histones will therefore make subtle contributions to overall average modification patterns. (PDF) [file pbio.1001075.s018.pdf]
