# Supplementary material for: Patterns and Mechanisms of Ancestral Histone Protein Inheritance in Budding Yeast
Source: PLoS Biol. 2011 Jun 7;9(6):e1001075. doi: 10.1371/journal.pbio.1001075 (PMC3110181; doi:10.1371/journal.pbio.1001075)
Supplement: Text S1 — Quantitative model for multigenerational histone dynamics. Detailed description of model shown in Figures 5 and 6. (PDF) [file pbio.1001075.s023.pdf]

Supplamentry

Quantitative model of histones inheritance

In this supplementary material we describe in details the mathematical model of nucleosome inheritance. With this model we try to estimate the cumulative effect of several dynamic processes on nucleosomes inheritance patterns, and isolate the effect of each process by itself.

Data processing

Raw sequencing data of HA and T7 libraries after the tag swap before release from arrest (0 generations), and at 1, 3, and 6 generations after release were uniquely mapped to the *S. cerevisiae* genome. We extracted nucleosome positions for the aggregated sequencing of the 3 generations samples using *Template-Filtering* (Weiner *et al.* 2009). The result of this step is a list of $M = 62844$ nucleosomes centers $[c_1, c_2, ..., c_M]$ and width. For each nucleosome we counted the number of supporting reads at each sample separately (4 generations X 2 sample each(HA/T7) = 8 samples) . Denote:

1. $x_{i,t}^{old}$ - Number of reads for nucleosome i in generation t with old tag
2. $x_{i,t}^{new}$ - Number of reads for nucleosome i in generation t with new tag
3. N_t^{old} - Total number of mapped reads in generation t with old tag
4. N_t^{new} - Total number of mapped reads in generation t with new tag

Retention model

We assume that three independent processes affect nucleosome inheritance within coding regions. First, nucleosomes can be replaced at any given time during the cell cycle, this leads to loss of HA tag. Second, nucleosomes can slide upstream or downstream within the coding region, this is ascribed to transcription or chromatin remodelers. Third, during replication nucleosome spread via reassociation, we assume that the diffusion has a Gaussian form with some standard deviation. The probability that a nucleosome will diffuse x bp away from it's previous position can be calculated using the probability density function (pdf) of the normal distribution.

$$f(x; \mu, \sigma^2) = \frac{1}{\sqrt{2\pi\sigma^2}} e^{-(x-\mu)^2/(2\sigma^2)} \quad (1)$$

To summarize, the model parameters are:

1. r_i - turnover rate, **nucleosome-specific** term for H3 replacement based on prior experimental results (Dion, 2007), with H3 replacement resulting in loss of HA.

2. μ_g - sliding factor, **gene-specific** parameter for lateral movement of nucleosomes ascribed to transcription.

3. σ - diffusion standard deviation, **global parameter** that describes the extent of nucleosome spreading via reassociation during replication.

The HA tags of a specific nucleosome i at generation t will spread to it's neighboring nuclesomes in generation $t + 1$ (retaining some HA to itself) according to the normal pdf function (1) centered around $c_i - \mu_g$ (it's center position shifted according to the gene-specific sliding parameter) and a global standard-deviation (σ). Let $\phi_{i,t}$ be the relative fraction of HA tag at nucleosome i in generation t, we can represent $\phi_{i,t+1}$ recursively using our free parameters with the following equation:

$$\phi_{i,t+1} = \sum_{j \text{ in } i\text{'s gene}} r_j \cdot \phi_{j,t} \cdot f(c_i, c_j + \mu_g, \sigma^2) \tag{2}$$

Where c_i and c_j are the centers of nucleosomes i and j and μ_g is the gene-specific sliding parameter of nucleosome i. The relative fraction of HA tag for a specific nucleosome is the sum of HA tag it inherited from the surrounding nucleosome in the previous generation, inheritance is proportional to the distance from each neighbor.

Likelihood function

$\phi_{i,t}$ represent the fraction of HA tag that each nucleosome retain up to generation t, but we can only measure the relative occupancy of nucleosomes within each sample - for a specific tag and generation. In addition we know that some fraction of the cells do not recombine the HA tag away, to consider for nonswitching cells we introduce an additional parameter. Let ϵ be the fraction of nonswitching cells, and let $\theta_{i,t}$ be the new HA relative quantity at each generation:

$$\theta_{i,t} = \epsilon + (1 - \epsilon)\phi_{i,t} \tag{3}$$

Nucleosome always retain ϵ of HA tag taken from the nonswitching cells and from the remaining $1 - \epsilon$ it retain $\phi_{i,t}$ (2). To finalize our model we need to take into account that we measure relative occupancy within each sample and that each nucleosome has a prior occupancy level to begin with. To do so, we assign a *wild-type* occupancy for each nucleosome (taken from previous work - Weiner *et al.* 2009) which we'll denote o_i, and we normalized for relative occupancy:

$$\varphi_{i,t}^{old} = \frac{o_i \cdot \theta_{i,t}}{\sum_{j=1}^{M} o_j \cdot \theta_{j,t}} \tag{4}$$

$\varphi_{i,t}^{old}$ is the relative occupancy of nucleosome i at generation t with HA tag. Respectively, we define the relative occupancy of T7 tag as:

$$\varphi_{i,t}^{new} = \frac{o_i \cdot (1-\theta_{i,t})}{\sum_{j=1}^{M} o_j \cdot (1-\theta_{i,t})} \tag{5}$$

Finally we assume that the actual reads count for each sample $[x_{1,t}^{old}, x_{2,t}^{old}, ..., x_{M,t}^{old}]$ distribute multinomial with N_t^{old} and $[\varphi_{1,t}^{old}, \varphi_{2,t}^{old}, \cdots \varphi_{M,t}^{old}]$:

1. $[x_{1,t}^{old}, x_{2,t}^{old}, \cdots, x_{M,t}^{old}] \sim M(N_t^{old}, [\varphi_{1,t}^{old}, \varphi_{2,t}^{old}, \cdots \varphi_{M,t}^{old}])$
2. $[x_{1,t}^{new}, x_{2,t}^{new}, \cdots, x_{M,t}^{new}] \sim M(N_t^{new}, [\varphi_{1,t}^{new}, \varphi_{2,t}^{new}, \cdots, \varphi_{M,t}^{new}])$

Using the multinomial distribution assumption we can write the log likelihood function for the complete data:

$$\begin{aligned} L(D) &= \sum_{t=1,3,6} \sum_{i=1}^{M} [x_{i,t}^{old} \cdot \log(\varphi_{i,t}^{old}) + x_{i,t}^{new} \cdot \log(\varphi_{i,t}^{new})] \\ &= \sum_{t=1,3,6} \sum_{i=1}^{M} [x_{i,t}^{old} \cdot \log(o_i \theta_{i,t}^{old}) + x_{i,t}^{new} \cdot \log(o_i \theta_{i,t}^{new})] - \\ & \sum_{t=1,3,6} [N_t^{old} \log(\sum_{j=1}^{M} o_j \theta_{j,t}^{old}) + N_t^{new} \log(\sum_{j=1}^{M} o_j \theta_{j,t}^{new})] \end{aligned} \tag{6}$$

The model free parameters are gene-specific sliding factors and the a global standard deviation (σ) for nucleosome spreading, we optimize these parameters to maximize the likelihood of experimental observations using coordinate gradient ascent until convergence.
